# A genome-wide association study using a Vietnamese landrace panel of rice (*Oryza sativa*) reveals new QTLs controlling panicle morphological traits

**DOI:** 10.1186/s12870-018-1504-1

**Published:** 2018-11-14

**Authors:** Kim Nhung TA, Ngan Giang KHONG, Thi Loan HA, Dieu Thu NGUYEN, Duc Chung MAI, Thi Giang HOANG, Thi Phuong Nhung PHUNG, Isabelle BOURRIE, Brigitte COURTOIS, Thi Thu Hoai TRAN, Bach Yen DINH, Tuan Nghia LA, Nang Vinh DO, Michel LEBRUN, Pascal GANTET, Stefan JOUANNIC

**Affiliations:** 1 LMI RICE, University of Montpellier, IRD, CIRAD, USTH, National Key Laboratory for Plant Cell Biotechnology, Agronomical Genetics Institute, Hanoi, Vietnam; 2 UMR DIADE, University of Montpellier, IRD, Montpellier, France; 3 CIRAD, UMR AGAP, University of Montpellier, INRA, Montpellier, France; 4 Plant Resource Center, Hanoi, Vietnam; 5 UMR LSTM, University of Montpellier, CIRAD, IRD, Montpellier, France; 6 Present address: Plant Genetics Laboratory, National Institute of Genetics, Mishima, Japan; 7 Present address: Department of Molecular Biology, Palacký University, Olomouc, Czech Republic

**Keywords:** Rice, GWAS, Panicle, Architecture, Yield, Image analysis, Vietnam

## Abstract

**Context:**

Yield improvement is an important issue for rice breeding. Panicle architecture is one of the key components of rice yield and exhibits a large diversity. To identify the morphological and genetic determinants of panicle architecture, we performed a detailed phenotypic analysis and a genome-wide association study (GWAS) using an original panel of Vietnamese landraces.

**Results:**

Using a newly developed image analysis tool, morphological traits of the panicles were scored over two years: rachis length; primary, secondary and tertiary branch number; average length of primary and secondary branches; average length of internode on rachis and primary branch. We observed a high contribution of spikelet number and secondary branch number per panicle to the overall phenotypic diversity in the dataset. Twenty-nine stable QTLs associated with seven traits were detected through GWAS over the two years. Some of these QTLs were associated with genes already implicated in panicle development. Importantly, the present study revealed the existence of new QTLs associated with the spikelet number, secondary branch number and primary branch number traits.

**Conclusions:**

Our phenotypic analysis of panicle architecture variation suggests that with the panel of samples used, morphological diversity depends largely on the balance between indeterminate vs. determinate axillary meristem fate on primary branches, supporting the notion of differences in axillary meristem fate between rachis and primary branches. Our genome-wide association study led to the identification of numerous genomic sites covering all the traits studied and will be of interest for breeding programs aimed at improving yield. The new QTLs detected in this study provide a basis for the identification of new genes controlling panicle development and yield in rice.

**Electronic supplementary material:**

The online version of this article (10.1186/s12870-018-1504-1) contains supplementary material, which is available to authorized users.

## Background

Rice (*Oryza sativa* L.) is one of the most important food crops for more than half of the world’s population and is also considered as a model species for grasses for important agronomic traits. Due to the rapid growth of the world’s population as well as increasing urbanization and climatic changes, higher or at least sustainable rice yield is urgently required to meet world food demands. Rice yield is a complex agronomic trait that is directly determined by three component traits: the number of panicles per plant, the number of grains per panicle and grain weight [1, 2]. While grain weight is determined by two components, grain size (length, width and thickness) and the degree of grain filling, the number of panicles is dependent on tillering ability. The number of grains per panicle is dependent on the panicle architecture, which consists of a series of branches of different orders: rachis, primary branches, secondary branches, potentially tertiary branches and finally spikelets. In rice, one spikelet meristem produces one floral meristem; therefore, the number of spikelet meristems will determine the number of grains per panicle.

By analysing the diversity of yield-related traits in rice, many studies aiming at creating new varieties with higher yield have been carried out [1]. Over several decades, quantitative trait locus (QTL) mapping using bi-parental populations of *O. sativa* has allowed the identification of QTLs related to characters such as panicle branching or size (e.g. *Gn1a*, *DEP1*, *IPA1*/*WFP*), grain weight or size (e.g. *GS3*, *GW2*, *qSW5*/*GW5*) and grain filling (e.g. *GIF1*), thus revealing a large number of genes governing yield components [1–3]. The identification of other QTLs has shown that yield potential can also be influenced indirectly by factors relating to the physiological state of the plant. An example is the *GPS* (*GREEN FOR PHOTOSYNTHESIS*) QTL that influences photosynthesis rate through the regulation of carboxylation efficiency [4, 5]. Some specific alleles of the genes corresponding to these yield-related QTLs were selected during domestication and/or breeding programs for high yields.

More recently, genome-wide association studies (GWAS), applied to large panels of rice varieties, emerged as a more powerful approach to increase coverage of natural variation and the number of significant loci, especially for complex agronomic traits [6]. The development of New Generation Sequencing (NGS)-based technologies allows the study of genotypically wide panels with a high density of markers covering the entire genome. GWAS analyses were conducted on different panels of rice accessions, leading to the description of genomic regions of interest related to various agronomic traits such as leaf traits, primary metabolism, plant height, flowering time, grain quality, grain size, grain coloration and physiological features, salt and drought tolerance, blast resistance, nematode tolerance, aluminium tolerance, and root system architecture [7–16]. These studies highlighted not only previously characterized QTLs but also numerous new QTLs and candidate genes, providing a valuable resource for rice genetics research as well as genetic markers for breeding [7, 17]. Recently, three GWAS studies were published relating to morphological components of panicle architecture using different panels of genotypes [18–20]. These analyses led to the identification of numerous genomic sites of interest. Only a fraction of these sites were associated with genes already known to be involved in panicle development, indicating that a number of as yet unknown factors may be involved in the determination of panicle architecture. Moreover, these analyses illustrated the impact of the environment on the identified genomic sites, supporting the view that panicle architecture depends not only on genetic diversity but also the environment.

In this study, as a means of further dissecting the morphological components of panicle architecture, a genome-wide association study of panicle morphological traits was performed on a panel of Vietnamese landraces using an image-based analysis tool [21, 22]. Vietnam possesses a huge diversity of traditional rice varieties due to its geographical situation and range of ecosystems [23]. Vietnamese resources still constitute a largely untapped and highly valuable genetic resource for local breeding programs. In addition, only a small fraction of the rice genetic diversity of Vietnam has been exploited in previous association studies based on worldwide sampling. The phenotypic analysis of the Vietnamese panel we used indicated that the number of secondary branches is the main contributor to variation in spikelet number per panicle. Several QTLs associated with spikelet number, secondary branch number and primary branch number were identified in the full panel as well as in the *indica* and *japonica* subpanels. These results will be useful for the exploitation of these varieties in local breeding programs and will provide new knowledge for understanding the complexity of the genetic basis of panicle architecture.

## Methods

### Plant material and genotyping

A population of 159 traditional rice varieties representing the diversity of *O. sativa* species in Vietnam and three reference varieties (Nipponbare, IR64 and Azucena) was used for GWAS. This population was obtained from a Vietnamese landrace core-collection established from 2014 and genotyped using 241 Diversity Array Technology (DArT) markers and 25,971 Genotyping By Sequencing (GBS)-derived Single Nucleotide Polymorphim (SNP) markers [22]. The population was structured into two sub-panels (93 *indica* accessions distributed within 6 sub-groups and 63 *japonica* accessions distributed within 4 sub-groups) and three admixed accessions that were determined using STRUCTURE software v2.3.4 [24]. The information related to these varieties is presented in Additional File 1: Table S1 and are detailed in [22].

### Phenotypic analysis

Phenotyping for GWAS analysis was performed in field conditions near Hanoi at the Plant Resource Center located at An Khanh, Hoai Duc (21° 00′ 01″ N and 105° 72′ 55″ E) and Van Giang Agricultural Station located at Van Giang, Hung Yen (20° 90′ 42“N and 105° 94’ 78” E) during the wet season of 2014 and 2015, respectively. In both field experiments, seeds were sown at the same period. After transplanting at 2 weeks after sowing, the varieties were grown in lowland conditions in 1 m^2^ plots of 25 plants each. The experimental design was an alpha-lattice with 2 replicates [25]. A block factor of 9 was introduced to check for possible environmental variations within replicates, a single block containing 18 accessions (i.e. 18 plots).

**Fig. 1:**
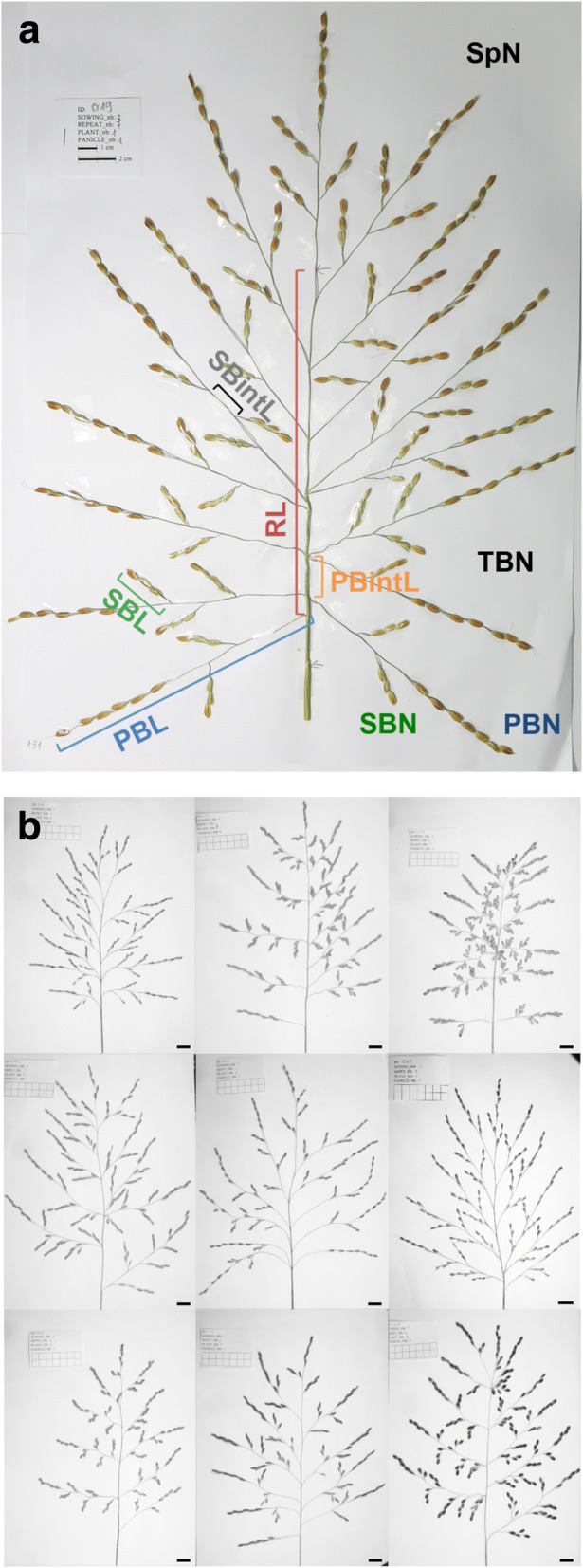
Panicle phenotyping. (**a**) Spread panicle with the quantified morphological traits using P-TRAP software. SpN: spikelet number; SBN: secondary branch number; PBN: primary branch number; TBN: tertiary branch number; SBintL: secondary branch internode average length; PBintL: primary branch internode average length; PBL: primary branch average length; SBL: secondary branch average length; RL: Rachis length; (**b**) Illustration of panicle architecture diversity in the Vietnamese landrace panel. Scale bar: 2 cm

About twenty days after the heading date, the three main panicles from three randomly chosen plants per variety per replicate were collected (i.e. 9 panicles/accession/replicate). Each panicle was spread out and fixed on white paper using tape. A total of 2916 panicles per season were analyzed using the P-TRAP software [21]. The quantified panicle traits included rachis length (RL), primary branch number (PBN), primary branch average length (PBL), primary branch internode average length (PBintL), secondary branch number (SBN), secondary branch average length (SBL), secondary branch internode average length (SBintL) and spikelet number (SpN) (Fig. 1a and Additional file 1: Table S1). Additional traits such as flowering date (FT), tiller number (TN) and efficient tiller number (eTN) were also recorded (Additional file 1: Table S1).

Panel statistics were calculated and statistical analysis of variance were performed using functions in R software. Analysis of variance (ANOVA) was applied, taking into consideration all factors (replicates, blocks within replicates and varieties) as fixed effects. Mean values were adjusted for variety, replicate and block factors. Trait heritability was computed using the variety effect based on the variance among phenotypic measurements between the two replicates of the population. A Shapiro test was used to determine if a dataset was well modeled by a normal distribution. The corrplot, ade4 and devtool packages were used to analyze the phenotypic correlation between traits and the organization of phenotypic variability, while linear regression analysis was used to obtain the coefficients of determination between PBN and SpN on the one hand, and SBN and SpN on the other hand. All statistical tests, clustering analysis, heat-map and principal component analyses (PCA) of the phenome dataset were performed using R software.

### Genome-wide association analysis

The entire genotypic panel, including 159 accessions with 26,212 markers, was analysed by applying a mixed linear model (MLM) according to [26]. The kinship matrix was established using the pairwise Identity by State (IBS) method proposed by Tassel v5.2.8 [27]. The structure matrix of the panels was determined by running a principal component analysis (PCA). The first two PCA axes were retained, respectively, and the scores of the accessions on these axes were used as the structure matrix. Three independent analyses were conducted using the full panel, the *indica* sub-panel and the *japonica* sub-panel respectively. Quantile-Quantile plots (QQ plots) and Manhattan plots with the threshold lines were performed using the QQman R package. A significance threshold of *P* < 0.001, based on the number of detected sites, was considered as corresponding to a significant association considering the globally low *p*-values. Only associations detected at *P* < 0.001 over the two years were considered as significant for further analysis.

Linkage disequilibrium (LD) between SNPs in the panel was evaluated by computing the r^2^ values between pairs of SNP markers using Tassel v5.2.8 on a chromosome basis. The LD heatmaps surrounding peaks in the GWAS were constructed using the LDheatmap R package and the candidate regions were defined by considering LD blocks with *r*^2^ > 0.6. On one hand, the significant markers of each LD block were used to define haplotypes using R software and then compare with the phenotype of each haplotype. On the other hand, genes and miRNA loci in LD regions were identified using MSU7, Rap_db database and mirBase v21 annotations (http://rice.plantbiology.msu.edu, http://www.gramene.org, http://www.mirbase.org, respectively). The qTARO (http://qtaro.abr.affrc.go.jp) and funRiceGenes databases (http://funricegenes.ncpgr.cn) were used to identify QTLs and to functionally characterize genes that overlapped with the LD regions associated with significant markers.

## Results

### Panicle architecture trait variation and heritability in the Vietnamese landrace panel

A Vietnamese landrace population [22] was phenotyped over two wet seasons (June to November 2014 and 2015) in lowland irrigated conditions in North Vietnam near Hanoi. Based on image analysis, a total of 9 panicle traits were scored (Fig. 1a, b and Additional file 1: Table S1). Each trial was replicated two times. Statistical analysis revealed that differences between the two replicates of the same year were not significant for any panicle traits (*P* > 0.001 at 1% threshold) (Table 1). Calculation of heritability scores showed that the scores of almost all panicle traits were similar and with high values (H^2^ > 0.85) for the two years, except for TBN which showed the lowest heritability score (0.21 in 2014 and 0.43 in 2015) and the highest variation with a coefficient of variation (CV) of the trait of over 35% with no significant genotypic effect (Table 1). This suggested that TBN depended more on environment than on genetic background. Consequently, this trait was not considered for the GWAS study. For other traits, secondary branch internode length and secondary branch number (SBintL, SBN) also displayed a wide phenotypic variation with a CV of over 25% while the CVs of other traits such as the length and number of primary branches and spikelets (SBL, PBL, PBN, SpN) were around 14–20% (Table 1).

**Table 1:** Phenotype variation and broad sense heritability (H^2^) for each panicle architecture trait in 2014 and 2015

	2014			2015		
Traits	Mean	CV	H^2^	Mean	CV	H^2^
SpN	180.9	21.78	0.91	216.1	22.57	0.89
PBN	12.3	15.02	0.94	13.6	14.23	0.93
SBN	34.1	26.18	0.91	40.7	25.47	0.88
TBN	0.1	154.45	0.21	0.6	193.97	0.43
PBL	11.3	13.64	0.94	16.5	14.07	0.93
SBL	2.6	15.01	0.91	3.6	14.92	0.96
RL	19.7	17.76	0.90	29.4	18.58	0.92
PBintL	1.8	23.17	0.93	2.4	22.04	0.96
SBintL	0.8	41.24	0.91	1.1	36.71	0.92

*SpN* spikelet number, *PBN* primary branch number, *SBN* secondary branch number, *TBN* tertiary branch number ,*PBL* primary branch average length, *SBL* secondary branch average length, *RL* Rachis length, *PBintL* primary branch internode average length, *SBintL* secondary branch internode average length, *Mean* Mean value of traits of 2 replicates, *CV* coefficient of variation of the trial

**Fig. 2:**
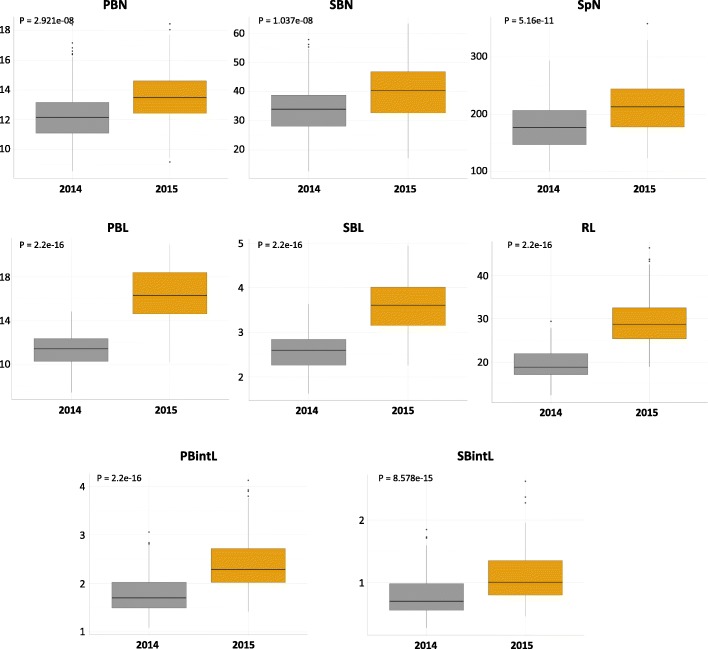
Boxplots of the distribution of panicle morphological traits in the two experiments. In gray and yellow are values for 2014 and 2015, respectively. The values of individuals for each class are presented in the y-axis (the values related to length are in cm). SpN: spikelet number; SBN: secondary branch number; PBN: primary branch number; SBintL: secondary branch internode length; PBintL: primary branch internode length; PBL: primary branch length; SBL: secondary branch length; RL: Rachis length. Statistical significance (i.e. *t* test *p* values) between the two years for the different panicle morphological traits is indicated

Comparison of panicle traits between the *indica* and *japonica* subpanels for each year indicated that the number of spikelets (SpN) as well as the number of secondary (SBN) and primary branches (PBN) were similar between the two subpanels (Additional file 2: Figure S1). This similarity indicated that population structure and phenotypic diversity were not correlated for these traits, leading in turn to the conclusion that this population was suitable for GWAS analysis of the traits in question. However, rachis lengths (RL) and primary internode average lengths (PBintL) within the *japonica* subpanel were found to be greater than for those of the *indica* subpanel (Additional file 2: Figure S1). In parallel, secondary branch internode average length (SBintL) was found to be shorter in the *japonica* subpanel than in the *indica* subpanel. Overall, *japonica* accessions in this population tend to have larger panicles than in the *indica* accessions. A comparison between the two years revealed a switch in the distribution of mean values for all traits, with significantly higher values in 2015 than in 2014 (Fig. 2). Overall, panicles in the 2015 experiment were larger and bore more spikelets than those of the 2014 experiment. However, traits relating to structure length were more impacted than those relating to structure numbers.

### Correlations between panicle architecture traits

**Fig. 3:**
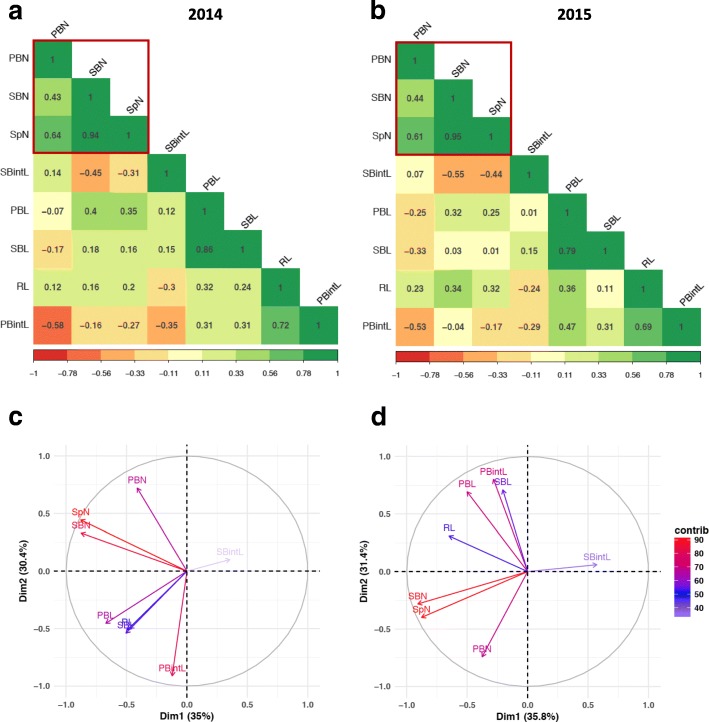
Correlation between panicle morphological traits of the full panel in the two experiments. (**a-b**) Correlation plots of panicle morphological traits for 2014 and 2015, respectively. (**c-d**) Principal component analysis (PCA) of panicle morphological traits for 2014 and 2015, respectively

Studies of phenotypic correlation and principal component analyses (PCA) between all traits revealed similar patterns over the two years (Fig. 3). Firstly, number-related traits SpN, PBN and SBN were highly positively correlated with each other (Fig. 3a and b), especially between SpN and SBN with a correlation value of over 0.94 for both years. The correlation between SpN and SBN was observed not only in the full panel, but also in the *indica* and *japonica* subpanels (Additional file 2: Figure S1 and Additional file 3: Figure S2). As secondary branches derive from primary branches, it is to be expected that a strong positive correlation is observed between the numbers of primary and secondary branches (Fig. 3a and b). Secondly, primary and secondary branch lengths were highly correlated with each other and were also correlated with rachis length albeit at a lower level, suggesting that these morphological components are determined by a globally regulated elongation process in the panicle. A low positive correlation was also observed between the average length of primary branches and the number of secondary branches, indicating that longer primary branches produce more secondary branches. In contrast, the length of the branches and their number were not correlated (i.e. PBN vs. PBL and SBN vs. SBL). In the same way, as expected, a negative correlation was observed between the numbers of primary or secondary branches and the average length of internodes (i.e. PBN vs. PBintL and SBN vs. SBintL). Finally, rachis lengths showed a positive correlation with all number-related traits (PBN, SBN and SpN) in addition to branch length (PBL and SBL) and PBintL. Additional traits were scored in the field such as flowering time (FT) in 2014 and 2015, and plant height (PH), tiller number (TN) and efficient tiller number (eTN) in 2015 only (Additional file 1: Table S1 and Additional File 3: Figure S2). A low positive correlation between FT and PH was observed over the two years. These two traits were positively correlated with panicle number traits but negatively correlated at a low level to panicle length traits over the two years. TN and eTN were highly correlated as expected but negatively correlated with panicle number traits and RL.

**Fig. 4:**
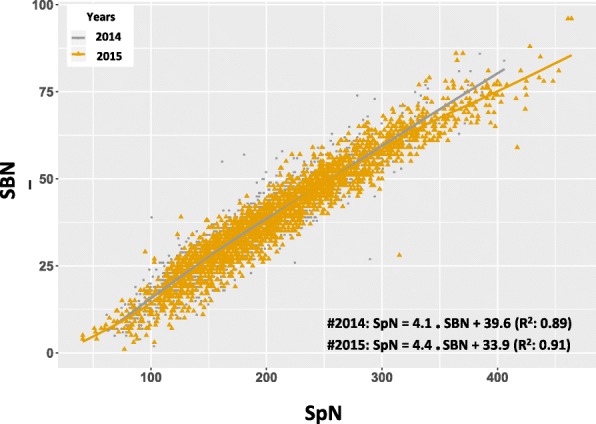
Correlation between spikelet number and secondary branch number in the full panel. In grey and yellow are values for 2014 and 2015, respectively. SpN: spikelet number. SBN: secondary branch number. The adjust R^2^ values of linear regression are indicated

Principal component analysis (PCA) revealed that the investigated traits could be resolved into two groups: number-related traits (PBN, SBN, SpN) and length-related traits (RL, PBL, SBL, PBintL, SBinL), with the first two PCA axes explaining about 60% of panicle trait variation (Fig. 3c and d). Spikelet number per panicle was the main trait contributing to the diversity observed in this population with a high correlation with SBN and intermediate correlation with PBN for the full panel as well as for the individual *indica* and *japonica* subpanels (Fig. 3c, d and Additional file 3: Figure S2). The adjusted R^2^ of linear regression analysis showed that the distribution of SBN explained 89 to 91% of SpN variation, while PBN variation explained only 37 to 42% of SpN variation (Fig. 4 and Additional file 4: Figure S3).

In addition, to establish a global phenotypic map of panicle traits for the two subpanels (*indica* and *japonica*), a clustering analysis was performed on the phenome dataset (Additional file 5: Figure S4). In a similar way to PCA, this analysis revealed distinct clades between the traits relating to structure numbers (PBN, SBN, SpN) and the traits relating to structure length (RL, PBL, SBL, PBintL, SBintL). Moreover this analysis confirmed that the clustering of the population based on panicle morphological traits was not strictly related to the genetic origin of the accessions (*indica* vs. *japonica*), with the exception of some clades (Additional file 5: Figure S4). This suggests that the full panel is suitable for GWAS for most of the traits studied here.

### GWAS for panicle architecture traits in the Vietnamese landrace panel

A set of 26,212 SNPs covering all the genome was selected from an initial genetic characterization of this Vietnamese landrace population [22]. This set was used on our panel of 159 accessions. We used a mixed linear model (MLM) to detect association signals for 8 traits: SpN, PBN, SBN, RL, PBL, SBL, PBintL and SBintL. In our study, the correlation between panicle traits and flowering date was not high (Additional file 3: Figure S2). Consequently, no correction/normalisation based on flowering date was carried out in our study. A total of 508 and 421 significant SNPs (*P* < 0.001) were detected for the 8 traits in 2014 and 2015 respectively (Additional file 6: Table S2). One hundred and five of these significant SNPs were stable over the two years. Manhattan plots and Quantile-Quantile plots for all traits over the two years are shown in Additional file 7: Figure S5. This analysis led to the identification of 29 QTLs detected for the same trait(s) over the two years (Table 2; see Additional file 8: Table S3 for the details). Corresponding genomic segments were defined according to the LD block size around the significant marker(s). For a single significant marker within a low LD block, a segment of 100 Kb around the marker (± 50 Kb) was arbitrarily selected.

**Table 2:** GWAS sites stable over the two years

QTL name	Trait	Chrom	Panel	Segment position (bp)	Sig. SNPs_ nb	Colocated genes		Colocated miRNA loci	
						Locus_id	Gene_symbol_Annotation	Locus_id	Annotation
QTL_1	PBN	1	ind	577,906–792,359	10			MI0008222	*osa-MIR531b*
QTL_2	SpN	1	FP	5,730,221–5,768,520	1				
QTL_3	PBintL	1	FP	7,051,706–7,151,706	1				
QTL_4	SpN	1	ind	8,812,823–8,951,604	3	LOC_Os01g15900*	RDD1		
QTL_5	PBN	1	FP & jap	22,974,971–23,332,164	7	LOC_Os01g40630*	LOG		
QTL_6	PBN & SpN	1	FP	23,846,573–23,946,573	1	LOC_Os01g42260	LEUNIG putative homologue		
QTL_7	SpN	1	ind	33,190,668–33,732,800	2	LOC_Os01g54620*	OsCesA4/BC7		
QTL_8	PBintL	1	FP_jap	34,237,359–34,435,745	1				
QTL_9	SBN & SpN	2	FP	16,621,984–17,305,751	9			MI0001688	*osa-MIR437*
QTL_10	PBN	2	FP & ind	23,998,460–24,128,723	3	LOC_Os02g39710*	OsCOL4	MI0000661	*osa-MIR156i*
QTL_11	PBN	3	FP	16,258,723–16,383,959	3				
QTL_12	SBL	3	FP	17,703,587–18,076,496	1				
QTL_13	SpN	3	ind	32,504,412–32,645,583	1			MI0000666	*osa-MIR160d*
QTL_14	RL & PBintL	4	ind	15,160,289–15,260,289	1				
QTL_15	RL	4	ind	24,257,299–24,356,022	4			MI0001102	*osa-MIR162b*
QTL_16	PBintL	4	FP	32,699,641–32,843,112	2	LOC_Os04g54900*	ILI1		
						LOC_Os04g55070	OsGA20ox2 putative homologue		
QTL_17	PBintL	6	FP	4,596,235–4,763,792	3				
QTL_18	PBintL	7	FP	18,924,787–19,100,034	1	LOC_Os07g32170*	OsSPL13		
QTL_19	PBN & PBL	8	FP & jap	5,221,963–5,450,178	1	LOC_Os08g09190	OsPILS2	MI0001133	*osa-MIR171b*
QTL_20	PBintL	8	FP	8,439,811–8,673,644	1				
QTL_21	PBintL	8	FP	18,989,868–19,215,198	8				
QTL_22	RL	9	FP	799,160–1,092,267	6				
QTL_23	PBintL	10	FP	15,641,392–15,741,392	1				
QTL_24	PBN	10	FP	17,498,080–18,064,123	5	LOC_Os10g33780*	TAW1		
						LOC_Os10g33940	OsARF22		
						LOC_Os10g33310*	OsiICK6		
QTL_25	RL	11	FP & jap	20,176,565–20,269,954	1	LOC_Os11g34460*	OsFKF1		
QTL_26	PBN	11	FP	21,409,189–21,444,141	1				
QTL_27	PBintL	11	ind	22,513,863–22,549,787	2				
QTL_28	SBintL	12	FP	5,748,587–5,859,468	3				
QTL_29	SBN	12	FP	15,423,955–15,784,175	1				

FP: full panel; *ind*: *indica* subpanel; *jap*: japonica subpanel; Chrom.: chromosome; Sig. SNPs_nb: number of significant SNPs; SBN: secondary branch number; PBN: primary branch number; SpN: spikelet number; SBintL: secondary branch internode length; PBintL: primary branch internode length; PBL: primary branch length; SBL: secondary branch length; RL: Rachis length; Locus-id: locus identification number in MSU7.0 (for coding genes) and mirBase v21 (for miRNAs); (*) Functionally characterized candidate genes in rice from OGRO and funRiceGenes databases (qtaro.abr.affrc.go.jp/ogro; funricegenes.ncpgr.cn)

**Fig. 5:**
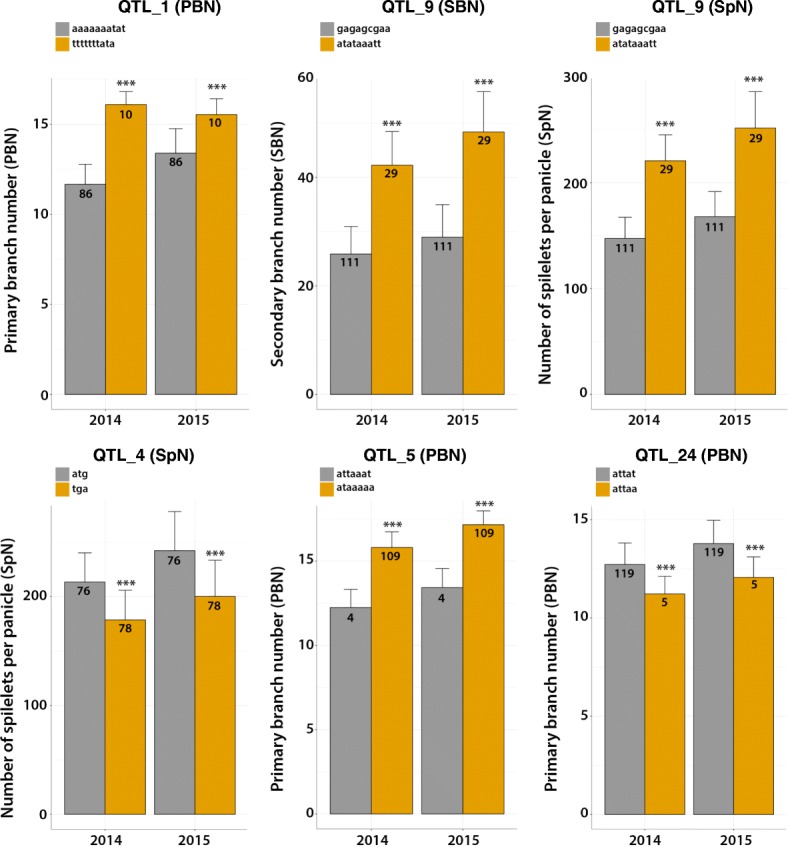
Allelic combination for QTL_1, QTL_4, QTL_5, QTL_9 and QTL_24 and their effect on the number of spikelets per panicle, secondary branch number and primary branch number. ***Indicates significant differences at *p* < 0.001 between allelic combination for each experiment. The two main haplotypes are represented. The number of accessions for each haplotype is indicated (n)

**Fig. 6:**
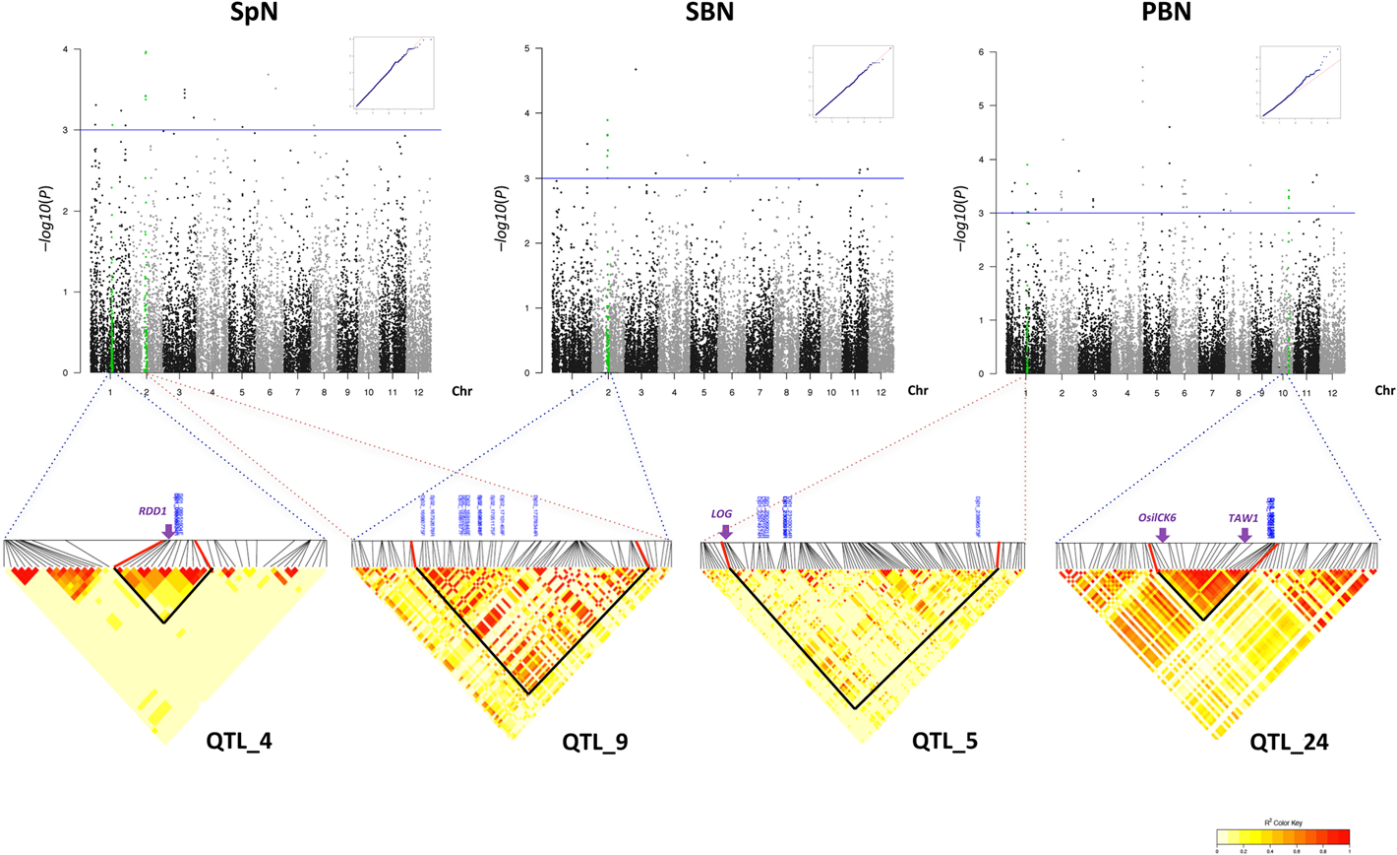
Genome-wide association study of SpN, SBN and PBN traits in the full panel. From top to bottom, QQ plot, Manhattan plot over the 12 chromosomes, and Linkage Disequilibrium (LD) heatmap surrounding the peak in the 2014 experiment for spikelet number (SpN) with QTL_4 and QTL_9 on chromosome 1 and 2 respectively, secondary branch number (SBN) with QTL_9 on chromosome 2 and primary branch number (PBN) for QTL_5 and QTL_24 on chromosome 1 and 10 respectively. Arrows indicate the position of functionally characterized genes. Red and bold back lines on the LD heat maps delimit the LD block for the GWAS peak. The significant SNPs are labelled in blue in the LD heatmap

A total of 6 stable QTLs were detected for SpN, 9 for branch number (8 for PBN, 1 for SBN), and 17 for length-related traits (1 for PBL, 1 for SBL, 4 for RL, 10 for PBintL and 1 for SBintL) (Table 2). Although a strong correlation was observed between SpN and branch number traits, especially SBN, only 2 of the 6 stable QTLs relating to SpN colocalized with other number-related traits (PBN and SBN) on chromosomes 1 and 2, respectively (QTL_6 and QTL_9, respectively in Table 2). However, QTL_6 was defined by a single significant marker, in contrast to QTL_9 which was defined by 9 significant markers (Table 2). Two other co-localizations were observed for RL and PBLintL on one hand, and for PBN and PBL on the other (QTL_14 and QTL_19, respectively in Table 2). No co-localizations were observed for PBL and SBL, nor for PBN and SBN. Of the 29 QTLs, 17 (59%) and 7 (24%) were detected specifically in the full panel and in the *indica* subpanel, respectively (Table 2). The 5 remaining QTLs were detected in the full panel and also in one of the two subpanels. Of the 13 QTLs relating to number traits detected over the two years, 8 were supported by more than one significant marker in the LD block (QTL_1, QTL_4, QTL_5, QTL_7, QTL_9, QTL_10, QTL_11 and QTL_24) (Table 2). Polymorphism combination analysis for these QTLs using the significant SNPs detected showed that the high SpN values were associated with the ATG haplotype in QTL_4, and with the ATATAAAT haplotype in QTL_9 (as SBN values for this QTL). The high PBN values were associated with the AAAAAAATAT, ATAAAAA and ATTAT haplotypes in the QTL_1, QTL_5 and QTL_24, respectively (Fig. 5). For QTL_7, QTL_10 and QTL_11, it was not possible to assign a specific haplotype to the high values of associated traits. QQ-plots and Manhattan plots over the 12 chromosomes for SpN, PBN and SBN are shown in Fig. 6, as well as LD heatmap plots for QTL_4, QTL_5, QTL_9 and QTL_24 illustrating the colocalization of GWAS sites and genes involved in panicle development and the colocalization of a GWAS site for both SpN and SBN.

In addition, twenty-one genomic regions were detected significantly over the two years but were associated with different traits (QTL_30 to QTL_50, Additional file 8: Table S3). In this context, 4 additional QTLs were detected for SpN, 10 for PBN, 4 for SBN, 3 for PBL, 3 for SBL, 10 for RL, 10 for PBintL and 5 for SBintL.

### Co-localized QTLs and functionally characterized genes

Most of the QTLs detected in this study co-localized with previously reported QTLs identified in bi-parental population studies and from other GWAS analyses relating to panicle and yield traits (Table 2; see Additional file 8: Table S3 for detailed information). Thirty QTLs of the 50 detected in our study overlapped with previously reported GWAS sites relating to panicle architecture (Additional file 8: Table S3) [18, 19]. Of the 25 co-localized sites common to our study and [18], only 3 sites shared comparable/similar traits (namely QTL_18 and QTL_38 for PBintL, and QTL_39 for PBN) (Additional file 8: Table S3). Similarly, only 4 sites co-localized with GWAS-derived QTLs from an *indica* panel phenotyped using the same image analysis software, but not for the same panicle trait [19] (Additional file 8: Table S3). In the later analysis, two QTLs displaying co-localization for SpN and SBN traits, one on chromosome 2 (q-9) and one on chromosome 11 (q-46), were reported but none of them co-localized with QTL_9 associated with the two traits in our study [19].

Co-localization with QTLs identified in bi-parental populations were observed for the same traits as for QTL_2 and QTL_13 for SpN trait and QTL_19 for the PBN trait (Additional file 8: Table S3). QTLs identified in bi-parental populations that related to other traits co-localized with GWAS-derived QTLs detected in our study. Among these, we observed co-localization of QTLs relating to the number of vascular bundles, root branching, chlorophyll content or flag leaf length with GWAS QTLs relating to primary branch number, spikelet number or rachis length (Additional file 8: Table S3). In the same way, co-localization was observed with GWAS QTLs relating to the root system that were identified using the same genetic population (Additional file 8: Table S3).

Of the 29 QTLs that displayed stability over the two years, 7 were associated with genes previously known to control panicle development and 3 additional QTLs were found to be associated with genes having an annotation suggestive of a role in the control of development (Table 2, Fig. 6 and Additional file 8: Table S3). In addition several annotated microRNAs were localized in the stable QTLs relating to panicle traits (Table 2; Additional file 8: Table S3). No candidate genes could be identified for QTL_9 (i.e. QTL associated to SpN and SBN traits on chromosome 2) on the basis of the functional annotation of the genes in this region, except for a monocot specific miRNA locus, *osa-MIR437* of unknown function (Table 2 and Fig. 6).

## Discussion

We conducted a phenotyping and the genome-wide association study of nine panicle morphological traits using 159 Vietnamese landrace accessions, to study the genetic basis of these traits and their putative interactions. The genotypic panel used had already been phenotyped for other traits (flowering time and root traits), and was deemed suitable for GWAS analysis as reported in [12, 22]. Our study allowed dissection of the various different components of panicle morphology relating to spikelet number variation and an evaluation of their contributions to this trait as well as to panicle size. This large-scale analysis was made possible by the use of an image-based software, which was especially useful for traits such as SBN, TBN and length traits [21]. A high heritability was observed for almost all morphological traits scored over the two years of field trials with the exception of TBN, as well as a good correlation between the two years, leading to the observation that the diversity of these traits was more under genetic control than under the influence of environmental conditions in this panel. In a similar way to results reported by [19] on an *indica* panel and by [20] on an *indica* and *japonica* panel, the main contributory component to variations observed in the Vietnamese panel is the variation of spikelet number (SpN), which is highly correlated with variation in secondary branch number (SBN) and, to a lesser extent, with variation in primary branch number (PBN). The high correlation of SBN with SpN was also reported in the context of bi-parental populations in a previous study [28]. The phenotypic variation of the panel was also analysed in the context of its genetic structure. The same phenotypic correlations were observed in the *indica* and *japonica* subpanels in our study. However, morphological differences were observed between these subpanels with, globally, larger panicle (i.e. high RL values) for the *japonica* than for the *indica* subpanel.

Overall, the level and nature of the observed correlations between traits show that variation of SpN relied more on number traits rather than on length traits in this panel and that the number of branches is globally independent of their length of the axils. This independence is coherent considering the kinetic of panicle development in which the branching phase (i.e. axillary meristem establishment and outgrowth) and the elongation phase (i.e. axil elongation) are disconnected with the large axil elongation occurring after establishment of axillary meristems and out-growth [29].

Despite a high correlation between the SpN and SBN traits, only a single GWAS site with co-location of SpN and SBN was observed on chromosome 2 (see Fig. 6), as well as a single site for SpN and PBN on chromosome 1 (Table 2). Several GWAS sites related to SpN did not co-localised with other morphological traits suggesting that SpN can be dependent on other traits that the ones quantified in this study. Similarly, distinct GWAS sites were observed for PBN and SBN suggesting that the mechanisms related to their establishment and their functioning are related to different genetic determinants. All the axillary meristems from rachis axil will contribute to primary branches. In contrast, the axillary meristem fate from a primary branch will balance between branch meristem (i.e. leading to a secondary branch) and spikelet meristem (i.e. leading to a lateral spikelet; see Fig. 1) [29]. Consequently, the intra-specific diversity for spikelet number per panicle is, at least for a part, related to the balance in meristem fate control in the second order of branching for indeterminate (i.e. branch meristem) vs. determinate (i.e. spikelet meristem) fate. This is in agreement with *O. sativa* mutant analyses leading to a model showing that the diversity of panicle complexity is related to fine-tuning of fate changes between branch and spikelet meristems [30].

Overall, this genome-wide association study led to the identification of 29 sites detected over two years for the same trait(s), for all the morphological traits quantified in this study. However, only 12 QTLs were associated to more than two significant markers in the corresponding LD blocks. This analysis would benefit from a larger density of SNPs for the association study and confirmation of the QTLs with a low number of associations. Overall, the QTLs detected in this study were characterized by a good stability between years but a low effect (i.e. low significance values) as also reported in other studies [19]. This may be explained by the additive effect of numerous determinants with no widespread major QTL, supporting the notion of a complex genetic basis for the individual morphological traits of the panicle.

Even if similar phenotypic features were observed between the different panels, no clear co-location of the GWAS sites for the same trait(s) was observed between our GWA study and those from [18–20], (with the exception of some common sites between our study and that reported by [18]). This may be a consequence of differences or specificities of the panels used in the four studies. The panel used in our study focused on Vietnamese landraces from both *indica* and *japonica* and not on elite accessions, in contrast with the genotypic panels used in the other studies mentioned. Another non-exclusive explanation is the effect of environmental factors on morphological panicle traits, bearing in mind that the various studies mentioned were conducted in different places and conditions around the world. Environmental impact is clearly illustrated by [19] with only two QTLs detected over the two experiments in different field conditions and in [20] with 18 QTLs detected in at least two conditions of the three tested. Our study over two years was conducted in similar (but not identical) environments and 29 QTLs corresponding to 105 significant SNPs were detected over the two years.

Some of the QTLs identified in our study were associated with genes that control panicle development and architecture such as the *TAWAWA1* (*TAW1*), *LONELY GUY* (*LOG*) and *RICE DOF DAILY FLUCTUATIONS 1* (*RDD1*) genes [31–33], thus illustrating the validity of our study. The later three genes were isolated via mutants displaying altered panicle architecture and until now had not been shown to contribute to natural variation of panicle traits such as PBN for *TAW1* and *LOG* (in QTL_24 and QTL_5 respectively) and SpN for *RDD1* (in QTL_4). The *TAW1* gene, encoding a transcription factor of the ALOG family, was reported as a regulator of spikelet fate acquisition influencing global architecture (and more specifically branch number) through the timing of acquisition of the determinate fate [31]. Expression of the *LOG* gene, encoding a cytokinin-activating enzyme that functions in the final step of bioactive cytokinin synthesis was associated with panicle meristem activity [32]. Interestingly, the *RDD1* gene, encoding a DOF transcription factor, which promotes nutrient ion uptake and grain yield, is regulated by photoperiod and circadian clock [33]. This gene, which is expressed in mesophyll, illustrates the possibility of there being an indirect effect (nutrient uptake) on panicle architecture in a source-sink relationship. Another example of an indirect effect on panicle development is illustrated by the *NARROW LEAF1* (*NAL1*) gene encoding a putative trypsin-like serine and cysteine protease, which affects vein patterning and polar auxin transport through cell proliferation [34]. The activity of *NAL1* is associated with a larger panicle and higher yield and the gene is co-localized with various QTLs [4, 35, 36]. In a similar way, several GWAS-derived QTLs detected in the present work co-localized with QTLs from bi-parental population studies relating to other traits, such as number of vascular bundles, root branching, chlorophyll content or flag leaf length. We also observed co-localization of some of our GWAS QTLs with some identified in relation to root system architecture using the same genetic population [12]. These co-localized genomic positions might represent physically related but independent determinants; however they might also be an indicator of panicle trait being indirectly affected by other traits, such as root and/or leaf structure, through a source-sink relationship at plant level.

Other stable QTLs were found to be associated with functionally characterized genes that had not yet been reported as being involved in panicle architecture control but which may have an indirect effect on yield. These genes are involved in the processes of cell division (e.g. *INHIBITORS OF CYCLIN-DEPENDENT KINASE 6, OsiICK6,* associated with PBN), flowering transition (e.g. *FLAVIN-BINDING KELCH REPEAT F-Box 1*, *OsFKF1,* associated with RL; *CONSTANS-LIKE 4*, *OsCOL4,* associated with PBN), brassinosteroid pathway (e.g. *INCREASED LEAF INCLINATION1, OsILI1,* associated with PBintL) or cellulose content (e.g. *OsCesA4/BC7* associated with SpN) [37–41]. Other genes that co-localized with QTLs remain to be functionally characterized but may be of interest on the basis of their annotation and/or homology to key genes in other species, such as two genes relevant to auxin signaling: *OsPILS2* and *OsARF22,* associated with PBN and PBL, corresponding respectively to a PIN-like and an Auxin Response Factor (ARF) gene [42, 43]; *LOC_Os01g42260*, associated with PBN and SpN, which encodes a putative homolog of LEUNIG (LUG), a transcriptional repressor in *A. thaliana* acting in floral organ identity specification [44]; and *LOC_Os04g55070* associated with PBintL and corresponding to a paralog of the *OsGA20ox2* / *SEMIDWARF 1* (*SD1*) gene involved in the gibberellin biosynthesis pathway associated with plant growth and development [45].

In addition to the annotated genes, some microRNA loci of interest were found to be located in GWAS sites. The microRNA *miR156* with a locus located in QTL_10 (PBN) is widely conserved in plants. It is known to be associated with panicle branching in rice through the targeting of transcripts of the SPL gene family, notably *OsSPL14*/*IDEAL PLANT ARCHITECTURE* (*IPA)* [46]. The microRNA *miR160* located in QTL_13 (SpN) is reported to target Auxin Response Factors (ARFs) transcripts, notably *OsARF18* which has pleiotropic effects on plant development and growth [47]. The microRNA *miR162*, which is widely conserved in plants*,* has a locus located in QTL_15 (RL). This microRNA is reported to be associated with the negative feedback regulation of Dicer-Like *DCL1* transcripts in Arabidopsis by microRNA-guided mRNA degradation [48]. The microRNA *miR171* targets transcripts of GRAS plant-specific transcription factor family associated with phase change from vegetative to reproductive development and shoot apical meristem maintenance in rice [49]. With the exception of *miR156*, none of these miRNAs has previously been implicated in the regulation of panicle development in rice, but on the basis of existing knowledge from rice and/or in other species, they may be good candidates. In addition to *miR156,* other microRNAs have been reported to be involved in panicle development, including *miR172* which regulates *APETALA2* (*AP2*) transcription factor genes [50, 51], *miR397* which regulates a laccase-like encoding gene [52] and *miR396* which regulates a *GROWTH REGULATING FACTOR 4* (*OsGRF4*) gene [53]. The later examples illustrate that microRNAs play an important role in panicle development and may be also considered as candidates for targeted breeding. In this context, it is interesting to note that studies of the genetic diversity of microRNA loci in *O. sativa* and its wild relative *O. rufipogon* revealed the presence of several loci showing significant signals for positive selection and/or potential domestication selection [54, 55].

As seen in other GWAS studies of panicle architecture [18–20], some important genes known to be involved in the regulation of inflorescence development such as *LAX PANICLE1* (*LAX1)*, *ABNORMAL PANICLE ORGANIZATION1* and *2* (*APO1*, *APO2*), *MONOCULM1* (*MOC1*), *DENSE AND ERECT PANICLE1* (*DEP1*) and *SPL14*/*IPA1* [56, 57] were not detected in our study. Most of the aforementioned genes were characterized from mutants and play a very important role in panicle growth and development [56, 57]. It is likely that any variation in these genes might tend to be eliminated in natural conditions if panicle growth were too severely affected. Alternatively sequence variations might occur at very low frequency and thus not be observed or filtered out in our analysis. Nevertheless a recent analysis revealed that a combination of favourable alleles for these genes might collectively produce a higher spikelet number in a specific panel, supporting the notion that there is a complex genetic network regulating this phenotypic feature [58].

Some stable QTLs related to number traits such as QTL_1 for PBN trait and QTL_9 for SpN and SBN traits were not observed to be associated with any functionally described genes. It will therefore be of interest to characterize the genes concerned in terms of their sequence and expression diversity in contrasting haplotypes of the relevant trait in order to identify candidates for functional validation. Another possible approach to investigate these statistically derived QTLs will be to develop bi-parental segregating populations for the validation and positional cloning of the QTL.

## Conclusions

Using image-based analysis software it was possible to dissect different components of panicle architecture, revealing that variations in spikelet number per panicle relate mostly to variations in secondary branch number. The 29 stable panicle trait QTLs identified by GWAS in the present study will provide new information on novel genetic determinants of panicle architecture. All the QTLs identified in our study were characterised by small effects (i.e. low *p*-values), supporting the notion of complex genetic interactions for the control of individual components of panicle architecture. The overall structure of the gene network governing panicle traits is still far from resolved and only some of the previously identified players may be associated with the diversity studied here. The additive effects of these individual factors should be taken into consideration for breeding by the use of pyramidal or multigenic genome editing approaches [58, 59].

## Additional files

Additional File 1: **Table S1.** Phenotypic and passport data of the 159 accessions of the Vietnamese rice landrace collection in 2014 and 2015. The indicated phenotypic data correspond to the average values for each trait per accession per year. Values relating to length are in cm. (XLS 157 kb)

Additional File 2: **Figure S1.** The phenotype variation of *indica* and *japonica* subpanels in 2014 and 2015. Primary branch number (PBN), secondary branch number (SBN), spikelet number (SpN), primary branch length (PBL), primary internode length (PBintL), secondary branch length (SBL), secondary internode length (SBintL), tertiary branch number (TBN) and rachis length (RL). The values relating to length are in cm. Statistical significance (*t* test *p* values) between the two subpanels for the different panicle morphological traits is indicated (PDF 378 kb)

Additional File 3: **Figure S2.** Correlation plots and PCA in the full panel, *indica* and *japonica* subpanels over the two years including flowering time (FT), plant height (PH), tiller number (TN) and efficient tiller number (eTN). (**a**) Correlation plots of the full panel in 2014 (left) and 2015 (right). (**b**) PCA of the full panel in 2014 (left) and 2015 (right). (**c**) Correlation plots of the *indica* (left) and the *japonica* (right) subpanels in 2014 (top) and 2015 (bottom). Primary branch number (PBN), secondary branch number (SBN), spikelet number (SpN), primary branch length (PBL), primary internode length (PBintL), secondary branch length (SBL), secondary internode length (SBintL), tertiary branch number (TBN) and rachis length (RL). (PDF 475 kb)

Additional File 4: **Figure S3.** Correlation between SpN and PBN traits in 2014 and 2015. In grey and orange are values for 2014 and 2015, respectively. The right panel indicates the value density for the two traits in 2014 (grey) and 2015 (orange). SpN: spikelet number. PBN: primary branch number. (PDF 123 kb)

Additional File 5: **Figure S4** Heatmap clustering of the accessions related to panicle morphological traits in 2014 and 2015. In red and blue are the values for *indica* and *japonica* subpanels, respectively. Primary branch number (PBN), secondary branch number (SBN), spikelet number (SpN), primary branch length (PBL), primary internode length (PBintL), secondary branch length (SBL), secondary internode length (SBintL), tertiary branch number (TBN) and rachis length (RL). (PDF 271 kb)

Additional File 6: **Table S2.** List of the significant markers from GWAS in 2014 and 2015 in the full panel, the *indica* and the *japonica* subpanels. The position (POS, in bp) in the chromosome (CHR) of each marker (based on MSU7.0 *O. sativa* Nipponbare reference genome) is indicated. The *p*-values associated to the traits are indicated. The values in bold are below 1. 10^− 4^. Primary branch number (PBN), secondary branch number (SBN), spikelet number (SpN), primary branch length (PBL), primary internode length (PBintL), secondary branch length (SBL), secondary internode length (SBintL), tertiary branch number (TBN) and rachis length (RL). (XLS 151 kb)

Additional File 7: **Figure S5.** QQ plots and Manhattan plots for panicle morphological traits for 2014 and 2015 in the full panel, *indica* and *japonica* subpanels. (**a, i**) Rachis length (RL); (**b, j**) primary branch number (PBN); (**c, k**) secondary branch number (SBN); (**d, l**) spikelet number (SpN); (**e, m**) primary branch length (PBL); (**f, n**) primary branch internode length (PBintL); (**g, o**) secondary branch length (SBL); (**h, p**) secondary branch internode length (SBintL) for 2014 and 2015, respectively (PDF 9355 kb)

Additional File 8: **Table S3.** Detailed information of the conserved GWAS sites over the two years (2014 and 2015). QTL_1 to QTL_29: conserved QTLs for the same trait(s) over the two years; QTL_30 to QTL_50: conserved QTL regions but for different traits between the two years; FP: full panel; SBN: secondary branch number; PBN: primary branch number; SpN: spikelet number; SBintL: secondary branch internode length; PBintL: primary branch internode length; PBL: primary branch length; SBL: secondary branch length; RL: Rachis length; PH: plant height; TN: tiller number; eTN: efficient tiller number. Locus-id: locus identification number in MSU7.0 (for coding genes) and mirBase v21 (for miRNAs); (*) Functionally characterized candidate genes in rice from OGRO and funRiceGenes databases (qtaro.abr.affrc.go.jp/ogro; funricegenes.ncpgr.cn). Genomic coordinates in red indicate genes that are located at a distance below 50 kb from the LD block of the corresponding QTL. (XLS 108 kb)

## Abbreviations

CV: Coefficient of variation
eTN: Efficient tiller number average
FT: Flowering time after sowing
GWAS: Genome-wide association study
LD: Linkage disequilibrium
PBintL: Rachis internode length average
PBL: Primary branch length average
PBN: Primary branch number per panicle
PCA: Principal component analysis
PH: Plant height average
QTL: Quantitative trait locus
RL: Rachis length average
SBintL: Primary branch internode length average
SBL: Secondary branch length average
SBN: Secondary branch number per panicle
SNP: Single nucleotide polymorphism
SpN: Spikelet number per panicle
TBN: Tertiary branch number per panicle
TN: Tiller number average
