# Supplementary figures and images for: A genome-wide association study using a Vietnamese landrace panel of rice (Oryza sativa) reveals new QTLs controlling panicle morphological traits
Source: BMC Plant Biol. 2018 Nov 14;18:282. doi: 10.1186/s12870-018-1504-1 (PMC6234598; doi:10.1186/s12870-018-1504-1)

2014

PBN

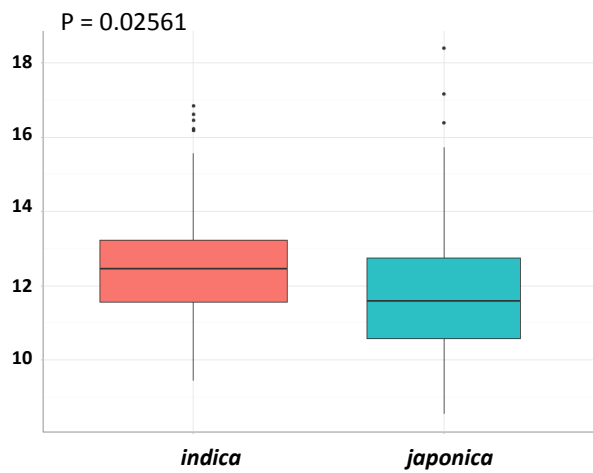

SBN

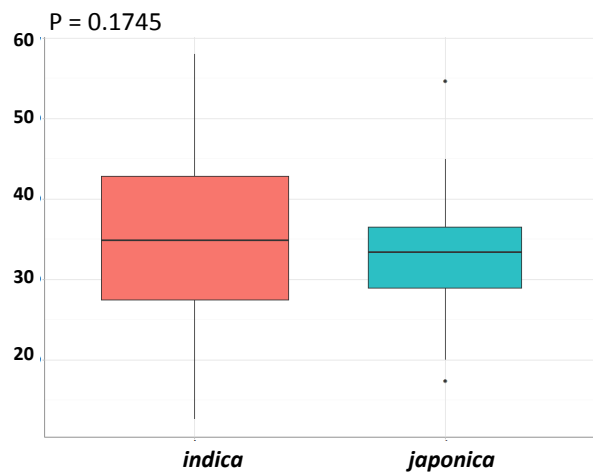

SpN

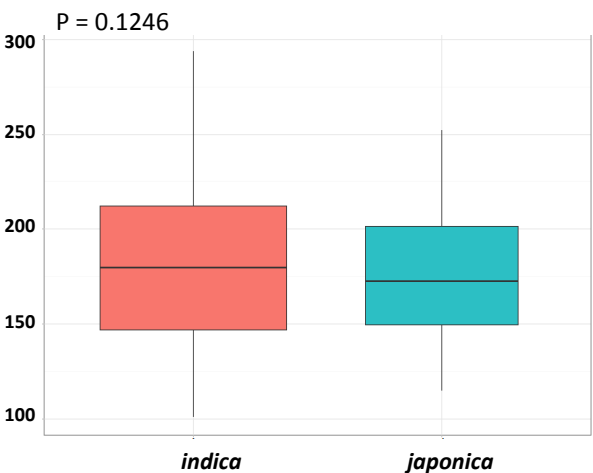

PBintL

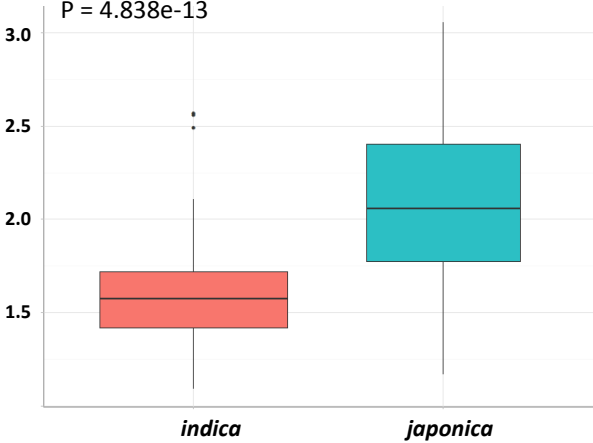

SBintL

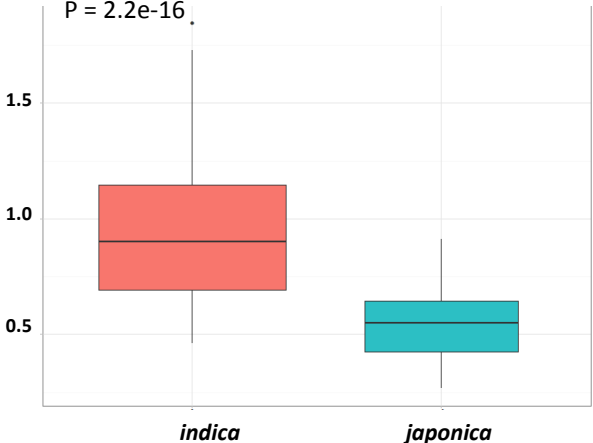

RL

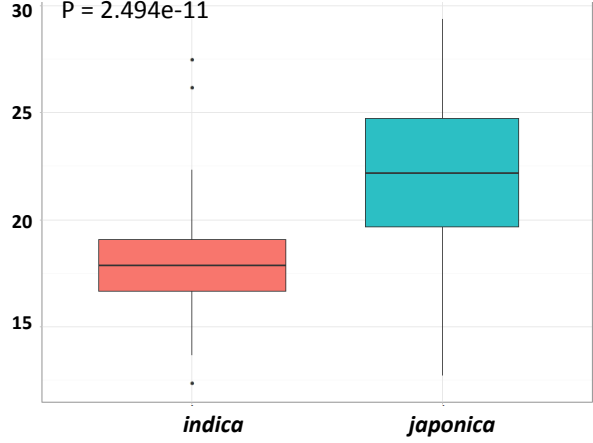

PBL

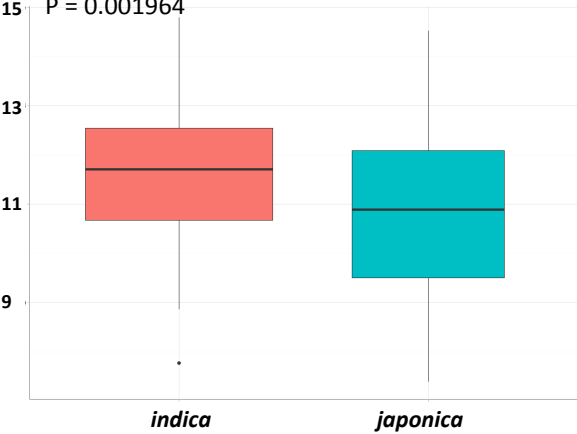

SBL

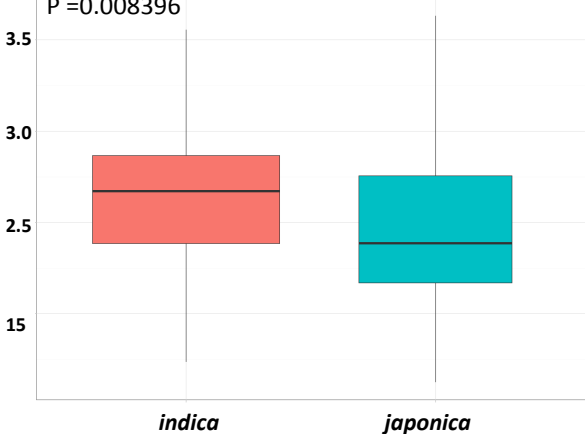

2015

PBN

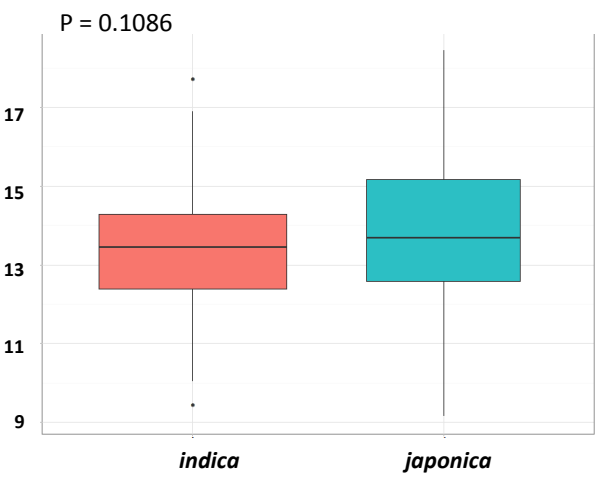

SBN

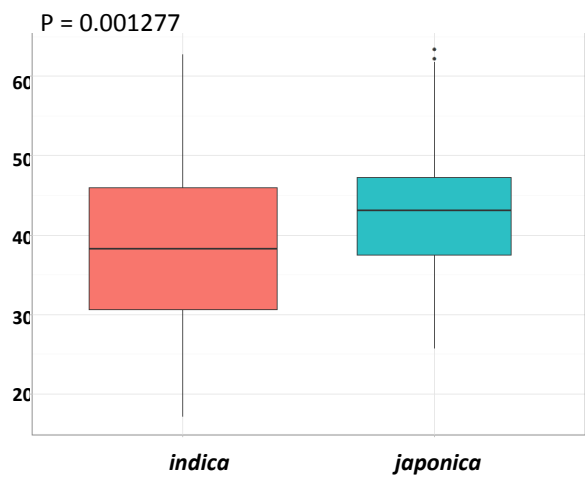

SpN

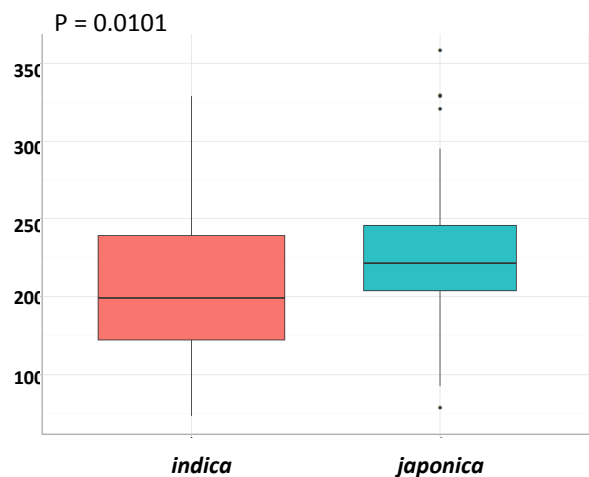

PBintL

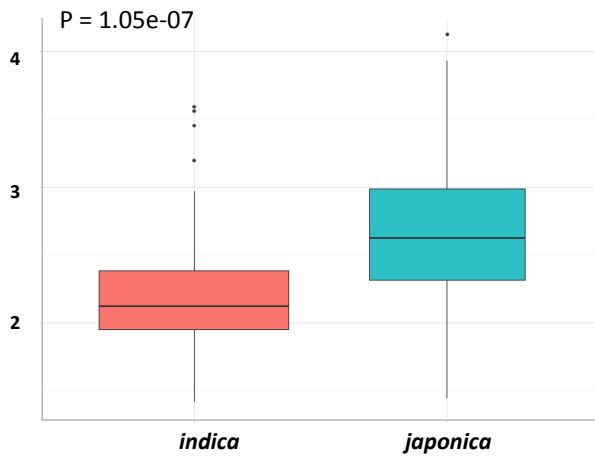

SBintL

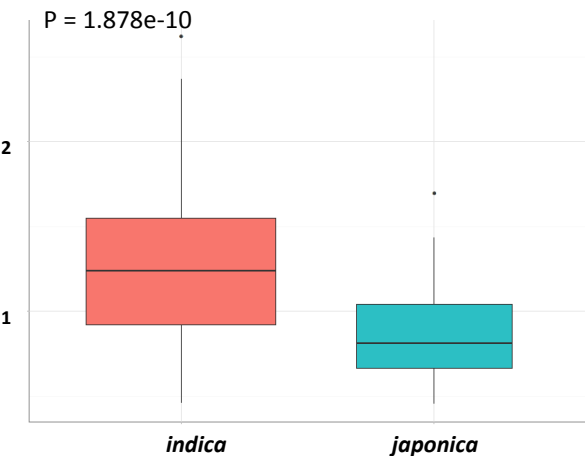

RL

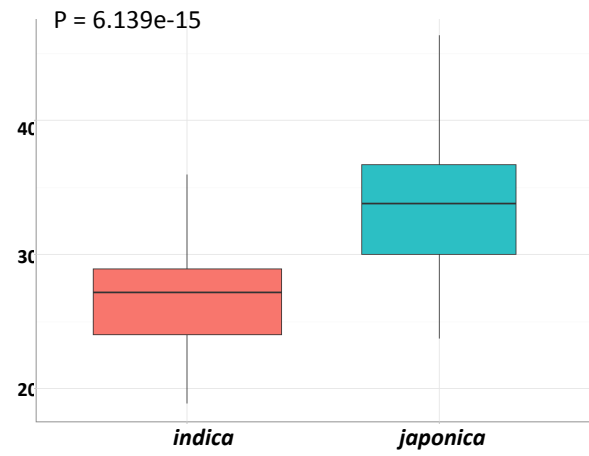

PBL

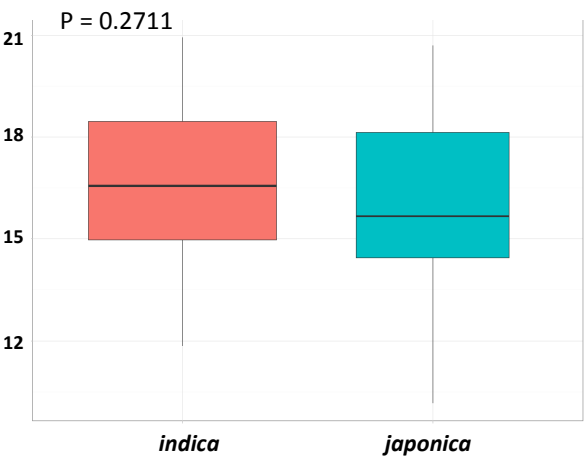

SBL

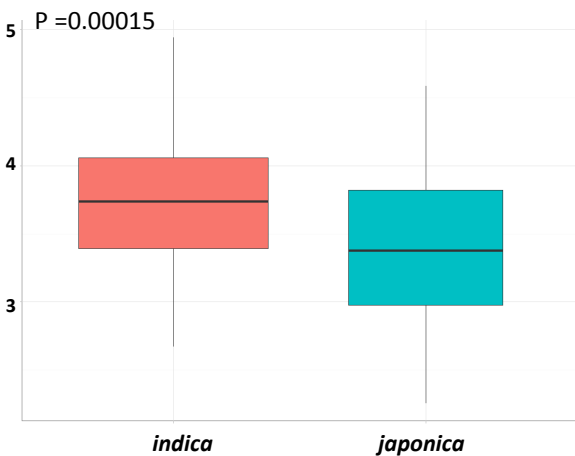

Supplement: Supplementary file 2 — Figure S1. The phenotype variation of indica and japonica subpanels in 2014 and 2015. Primary branch number (PBN), secondary branch number (SBN), spikelet number (SpN), primary branch length (PBL), primary internode length (PBintL), secondary branch length (SBL), secondary internode length (SBintL), tertiary branch number (TBN) and rachis length (RL). The values relating to length are in cm. Statistical significance (t test p values) between the two subpanels for the different panicle morphological traits is indicated (PDF 378 kb) [file 12870_2018_1504_MOESM2_ESM.pdf]

Full panel (2014)

Full panel (2015)

A

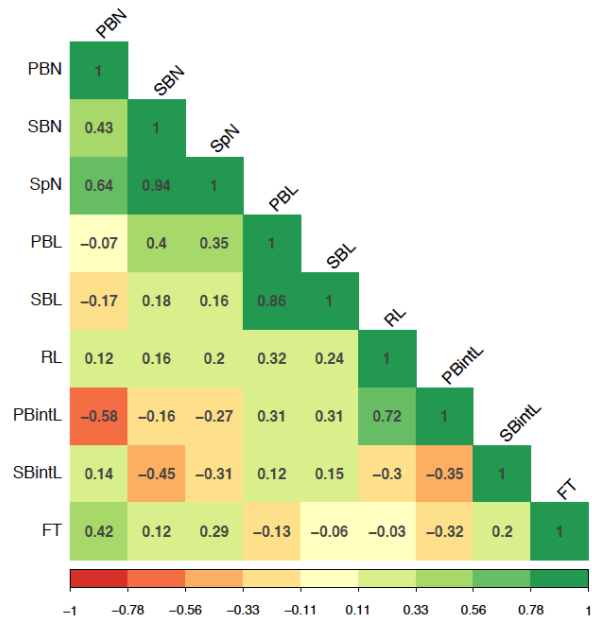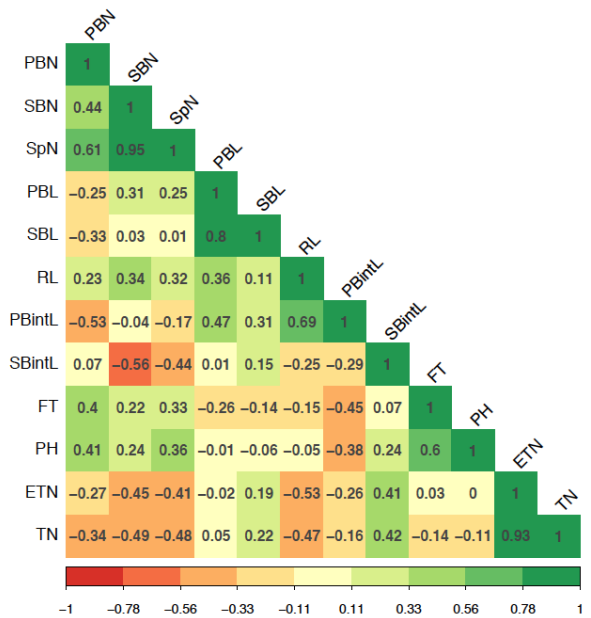

B

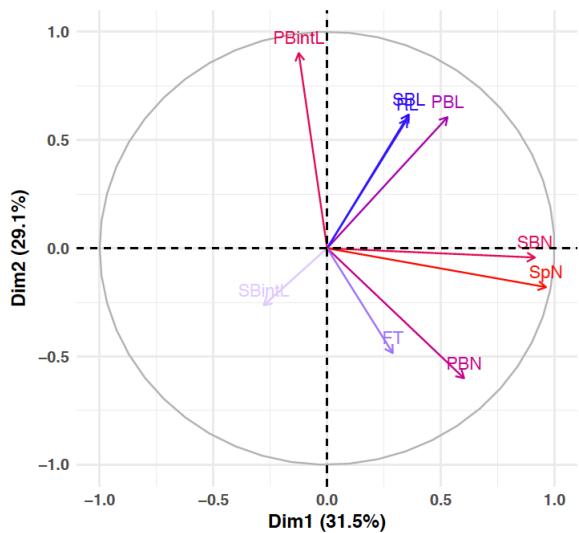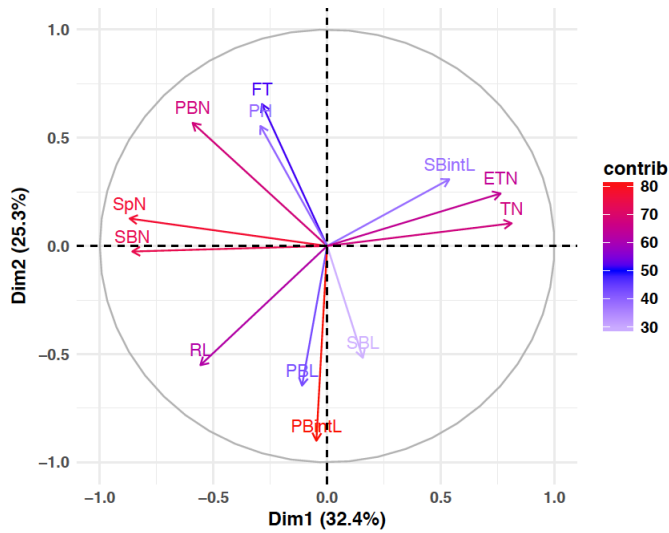

C

*indica* panel (2014)

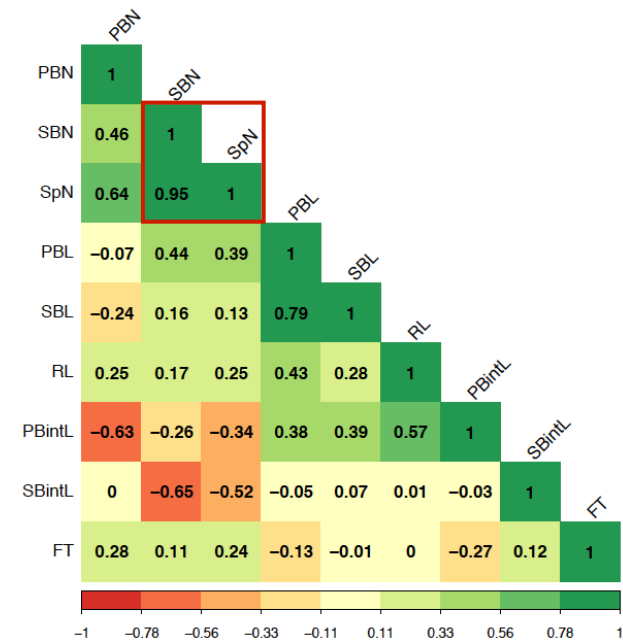

*japonica* panel (2014)

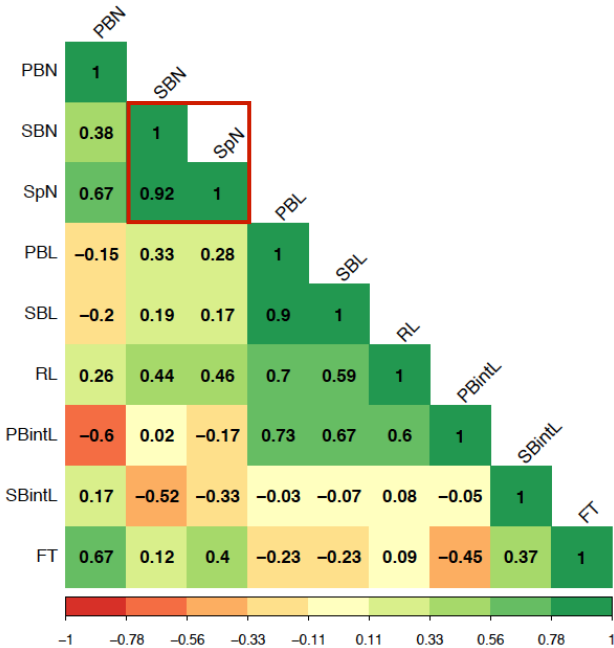

*indica* panel (2015)

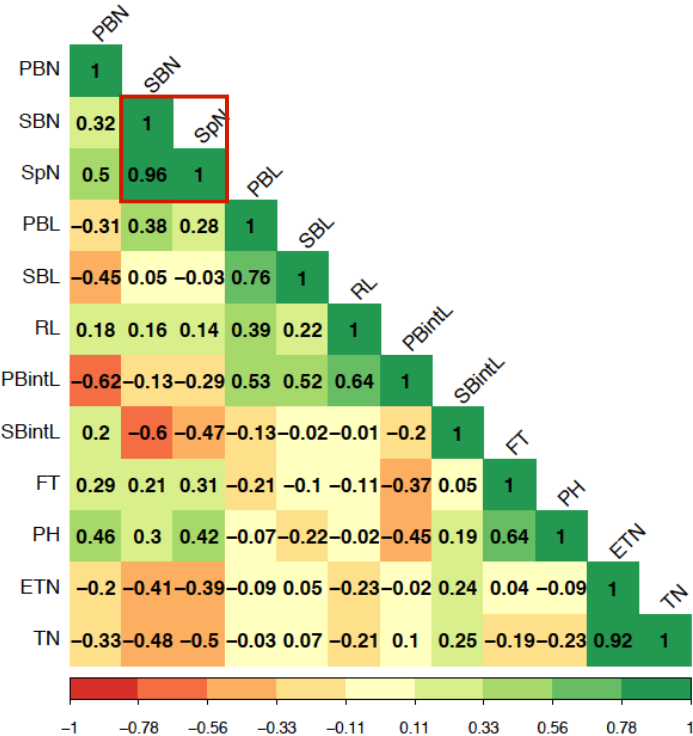

*japonica* panel (2015)

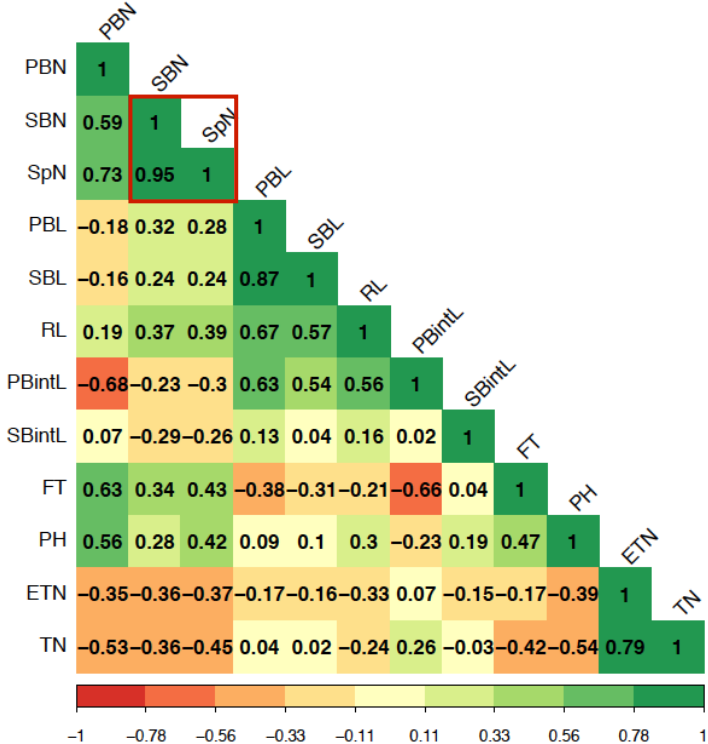

Supplement: Supplementary file 3 — Figure S2. Correlation plots and PCA in the full panel, indica and japonica subpanels over the two years including flowering time (FT), plant height (PH), tiller number (TN) and efficient tiller number (eTN). (a) Correlation plots of the full panel in 2014 (left) and 2015 (right). (b) PCA of the full panel in 2014 (left) and 2015 (right). (c) Correlation plots of the indica (left) and the japonica (right) subpanels in 2014 (top) and 2015 (bottom). Primary branch number (PBN), secondary branch number (SBN), spikelet number (SpN), primary branch length (PBL), primary internode length (PBintL), secondary branch length (SBL), secondary internode length (SBintL), tertiary branch number (TBN) and rachis length (RL). (PDF 475 kb) [file 12870_2018_1504_MOESM3_ESM.pdf]

## Correlation between SpN and PBN traits in 2014 and 2015

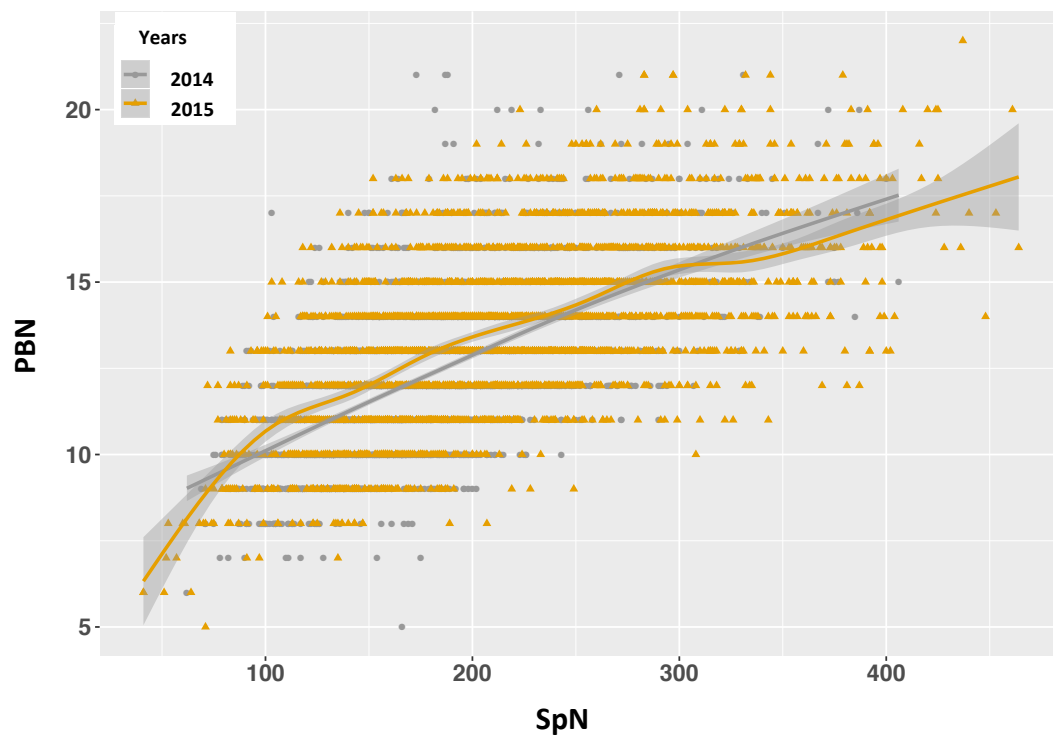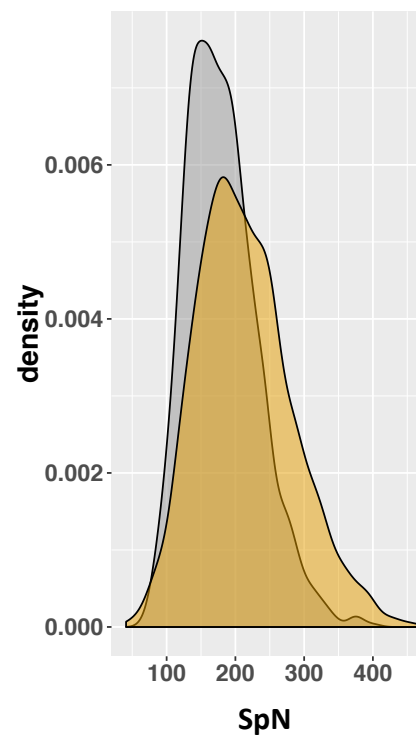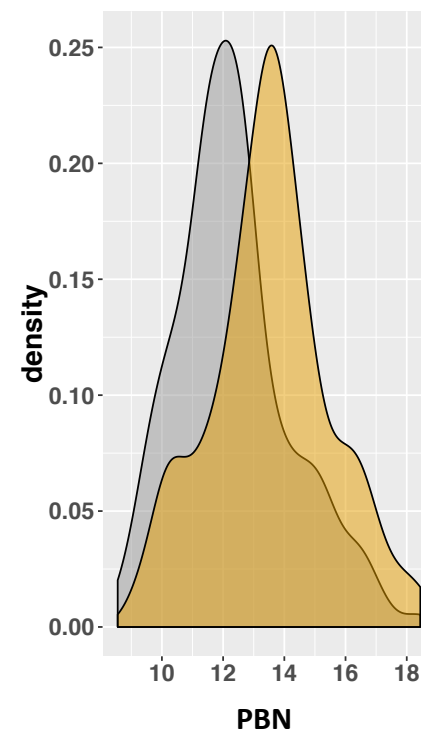

Supplement: Supplementary file 4 — Figure S3. Correlation between SpN and PBN traits in 2014 and 2015. In grey and orange are values for 2014 and 2015, respectively. The right panel indicates the value density for the two traits in 2014 (grey) and 2015 (orange). SpN: spikelet number. PBN: primary branch number. (PDF 123 kb) [file 12870_2018_1504_MOESM4_ESM.pdf]

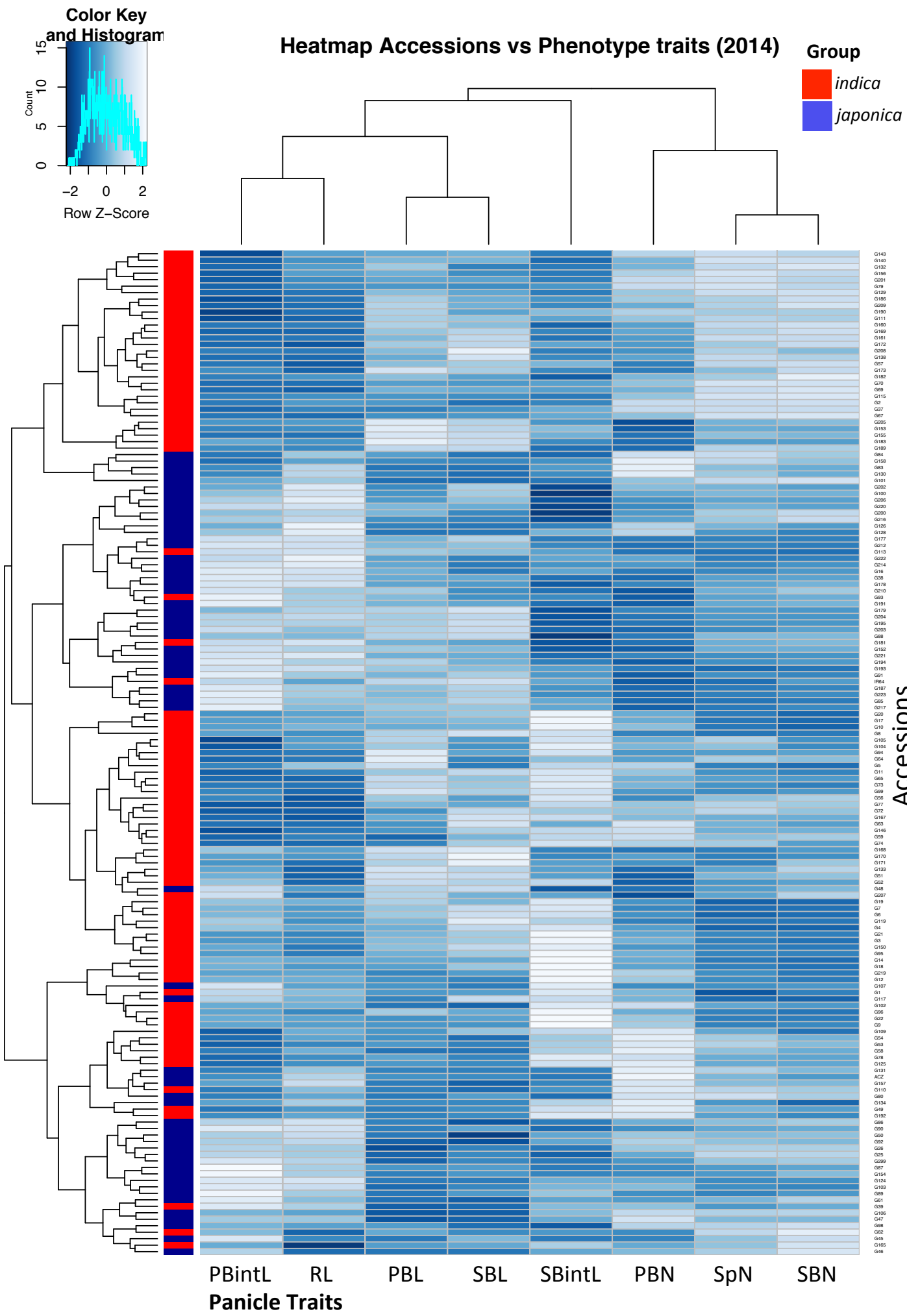

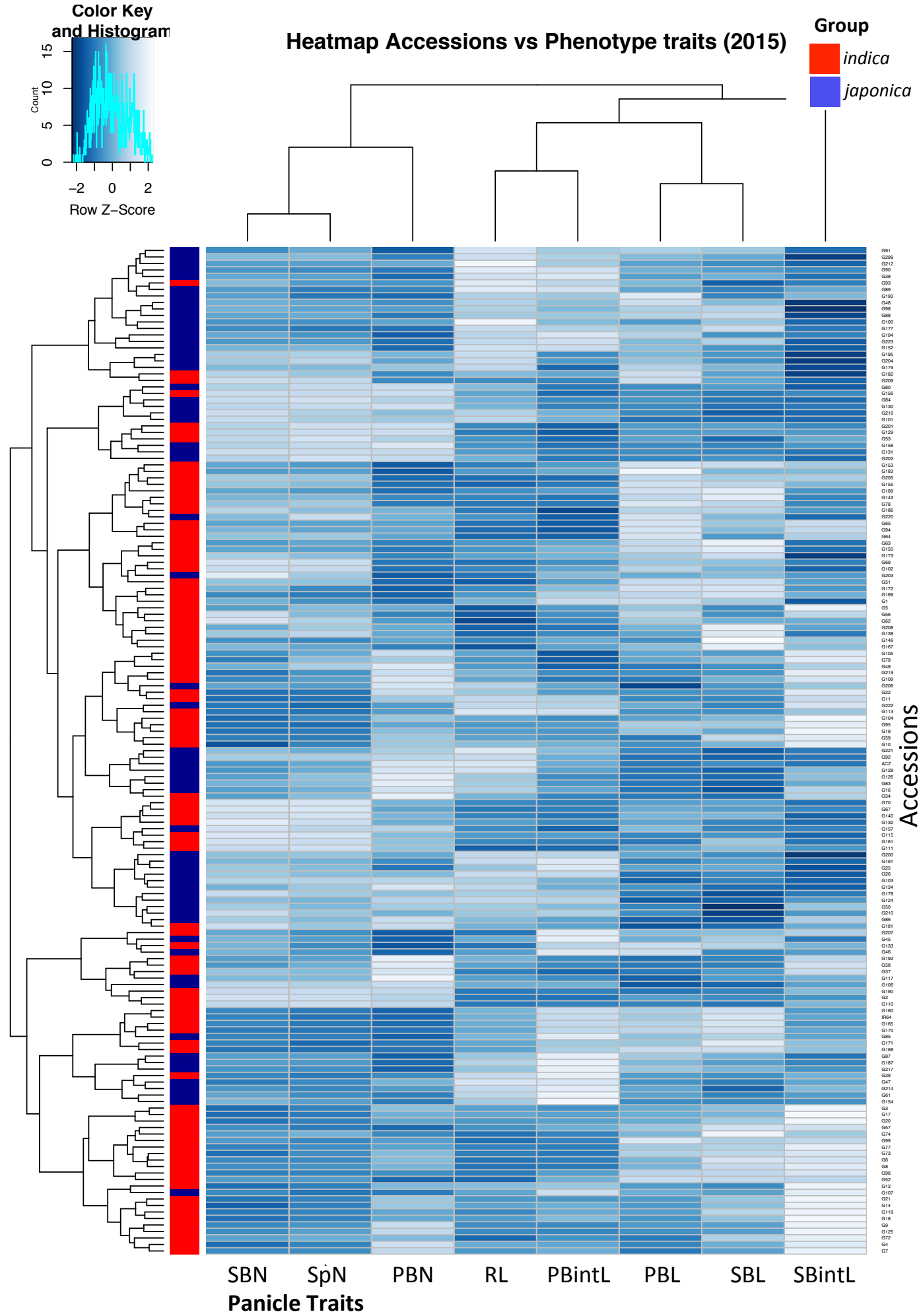

Supplement: Supplementary file 5 — Figure S4 Heatmap clustering of the accessions related to panicle morphological traits in 2014 and 2015. In red and blue are the values for indica and japonica subpanels, respectively. Primary branch number (PBN), secondary branch number (SBN), spikelet number (SpN), primary branch length (PBL), primary internode length (PBintL), secondary branch length (SBL), secondary internode length (SBintL), tertiary branch number (TBN) and rachis length (RL). (PDF 271 kb) [file 12870_2018_1504_MOESM5_ESM.pdf]

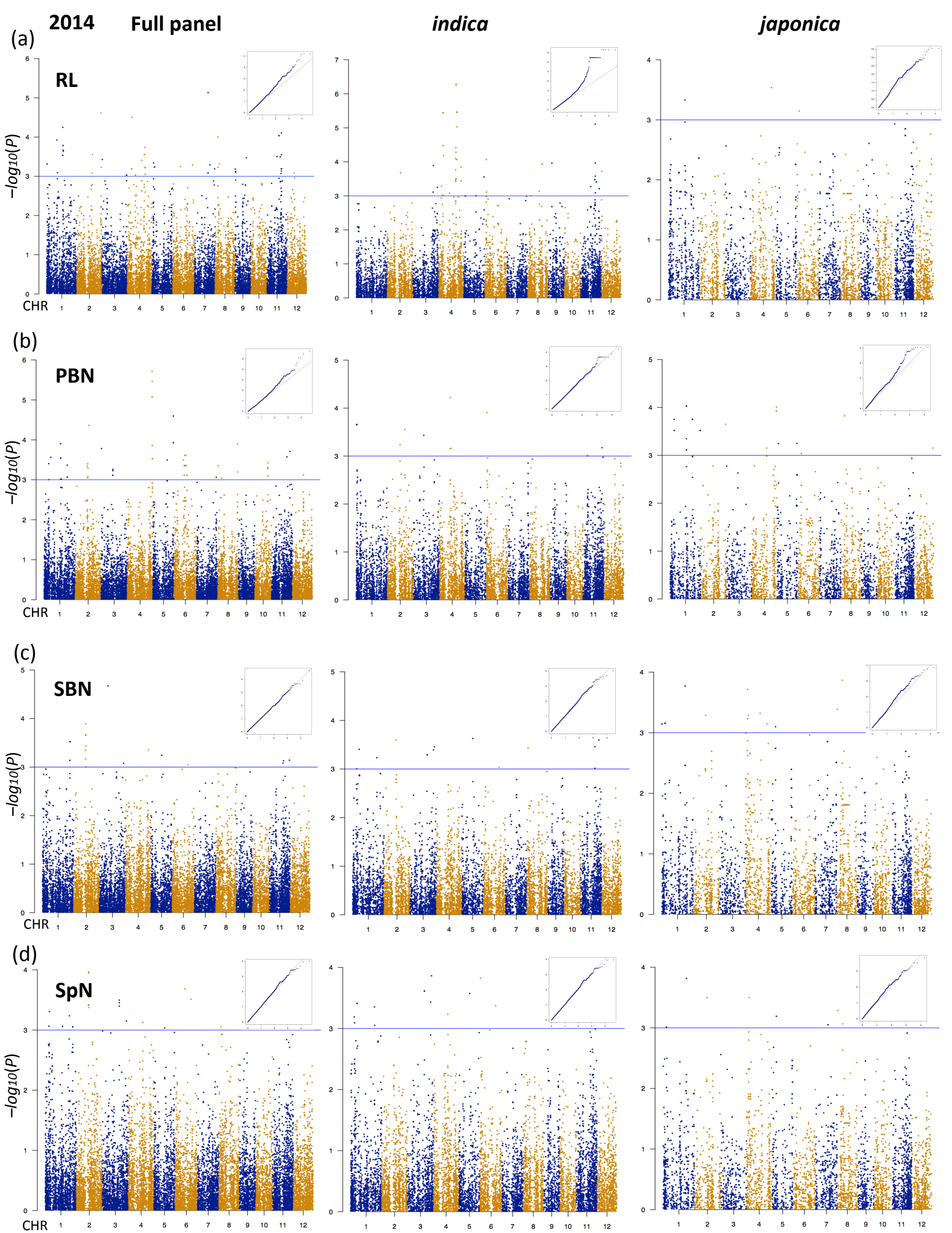

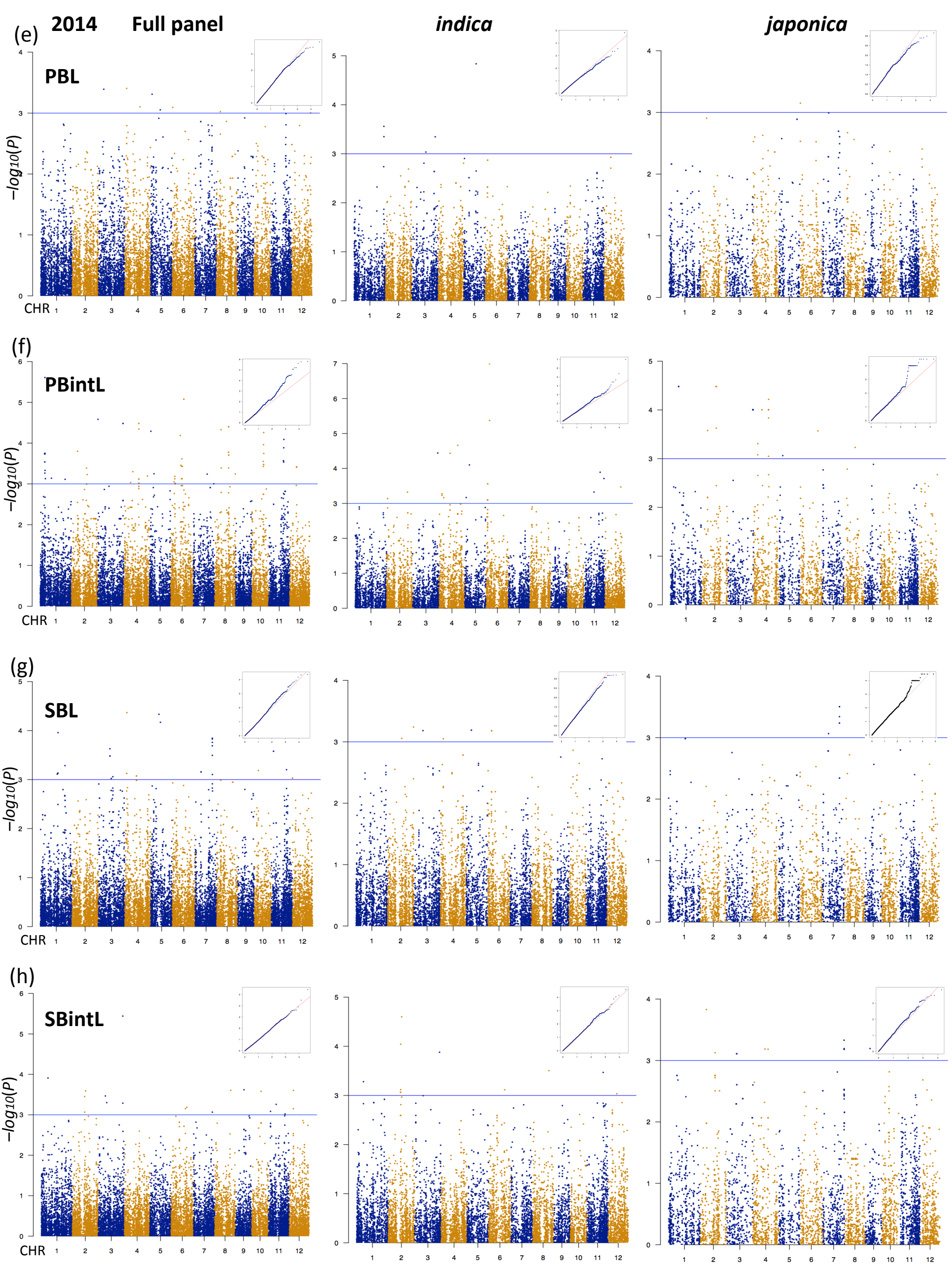

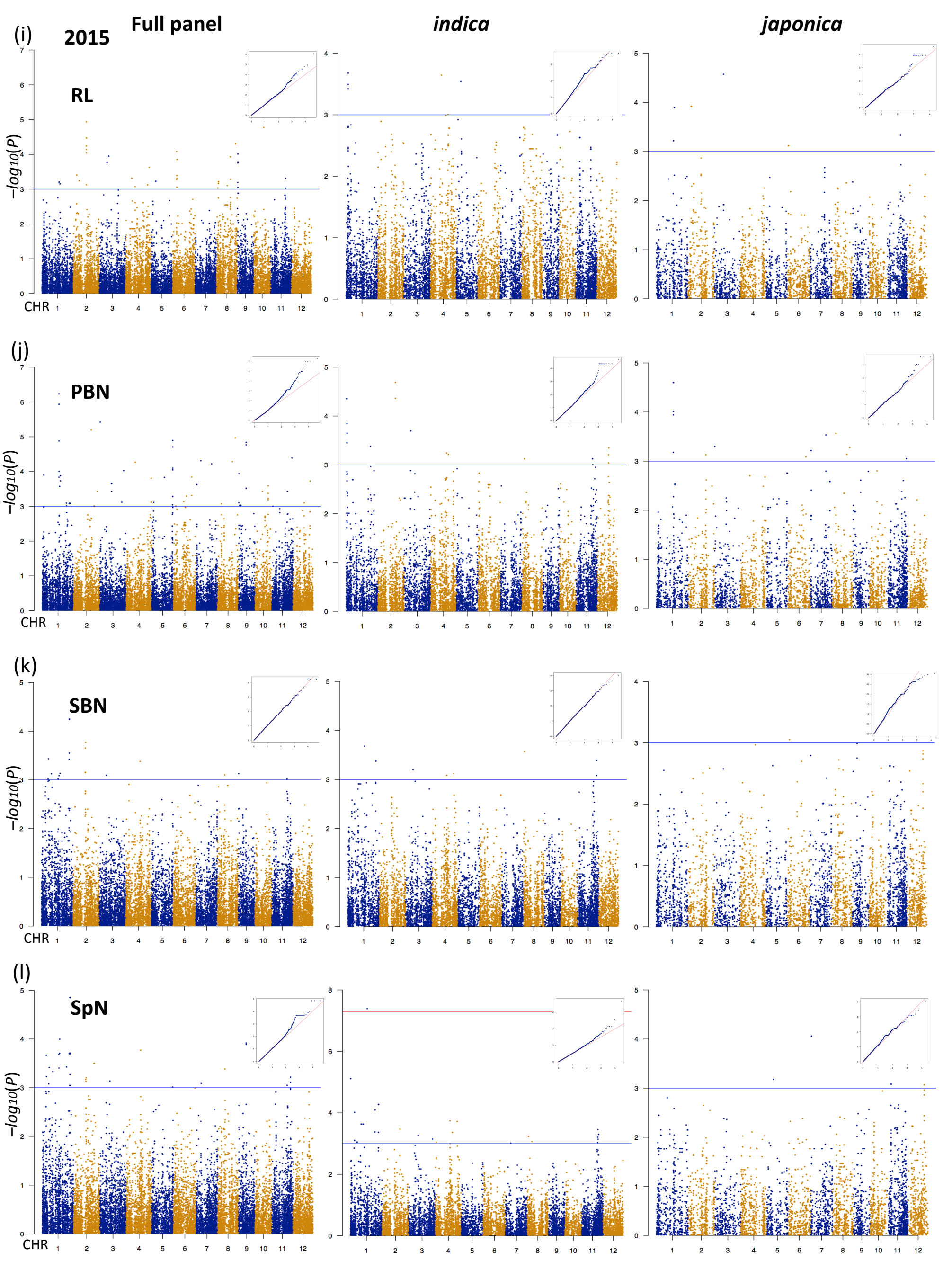

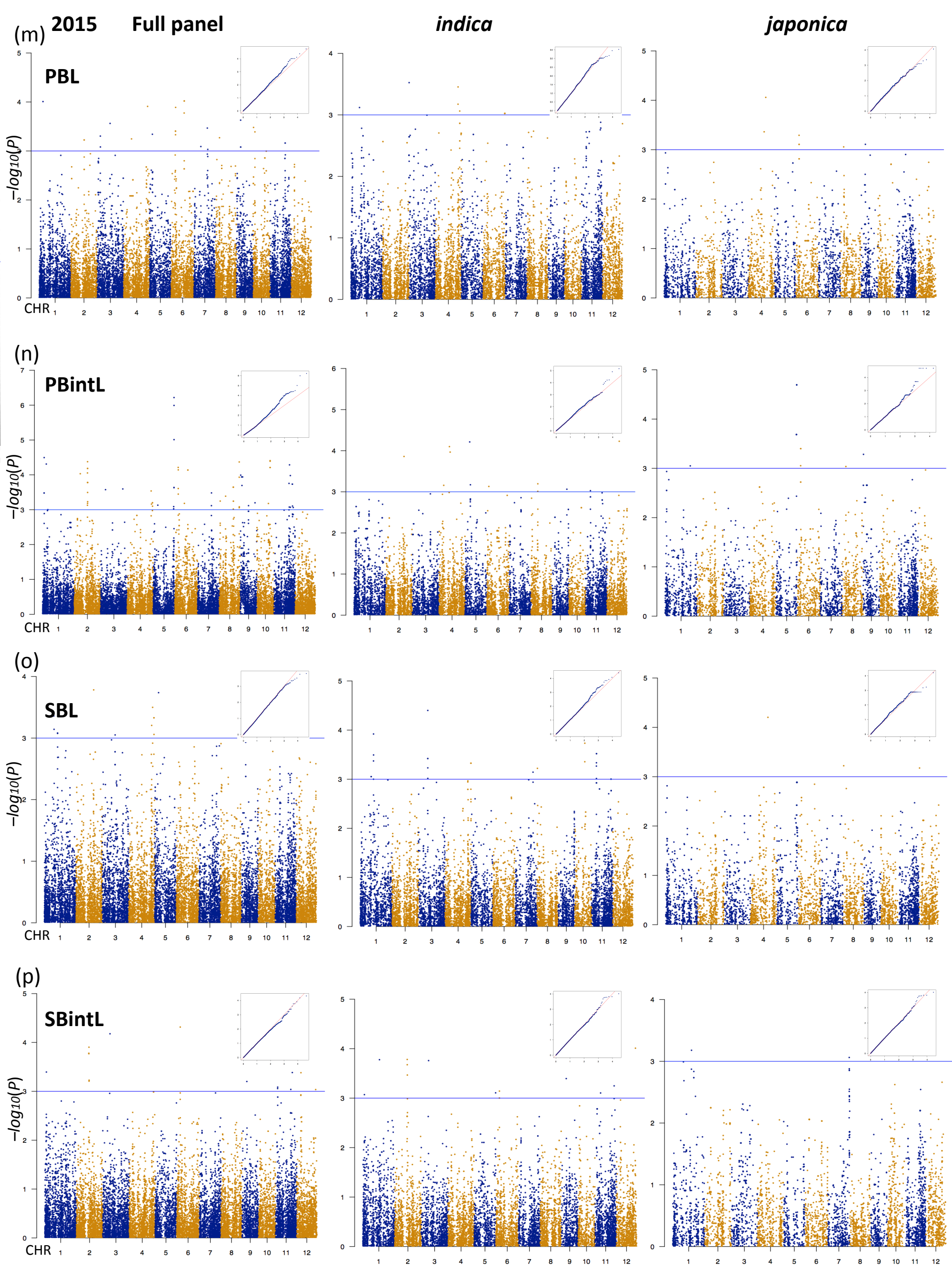

Supplement: Supplementary file 7 — Figure S5. QQ plots and Manhattan plots for panicle morphological traits for 2014 and 2015 in the full panel, indica and japonica subpanels. (a, i) Rachis length (RL); (b, j) primary branch number (PBN); (c, k) secondary branch number (SBN); (d, l) spikelet number (SpN); (e, m) primary branch length (PBL); (f, n) primary branch internode length (PBintL); (g, o) secondary branch length (SBL); (h, p) secondary branch internode length (SBintL) for 2014 and 2015, respectively (PDF 9355 kb) [file 12870_2018_1504_MOESM7_ESM.pdf]
